# A New Zebrafish Model of Oro-Intestinal Pathogen Colonization Reveals a Key Role for Adhesion in Protection by Probiotic Bacteria

**DOI:** 10.1371/journal.ppat.1002815

**Published:** 2012-07-26

**Authors:** Olaya Rendueles, Lionel Ferrières, Maxence Frétaud, Evelyne Bégaud, Philippe Herbomel, Jean-Pierre Levraud, Jean-Marc Ghigo

**Affiliations:** 1 Institut Pasteur, Unité de Génétique des Biofilms, Département de Microbiologie, Paris, France; 2 Institut Pasteur, Unité Macrophages et Développement de l'Immunité, Département de Biologie du Développement, Paris, France; 3 CNRS, URA2578, Paris, France; 4 Institut Pasteur, Centre de Ressources Biologiques de l'Institut Pasteur, Paris, France; Institut Pasteur, France

## Abstract

The beneficial contribution of commensal bacteria to host health and homeostasis led to the concept that exogenous non-pathogenic bacteria called probiotics could be used to limit disease caused by pathogens. However, despite recent progress using gnotobiotic mammal and invertebrate models, mechanisms underlying protection afforded by commensal and probiotic bacteria against pathogens remain poorly understood. Here we developed a zebrafish model of controlled co-infection in which germ-free zebrafish raised on axenic living protozoa enabled the study of interactions between host and commensal and pathogenic bacteria. We screened enteric fish pathogens and identified *Edwardsiella ictaluri* as a virulent strain inducing a strong inflammatory response and rapid mortality in zebrafish larvae infected by the natural oro-intestinal route. Using mortality induced by infection as a phenotypic read-out, we pre-colonized zebrafish larvae with 37 potential probiotic bacterial strains and screened for survival upon *E. ictaluri* infection. We identified 3 robustly protective strains, including *Vibrio parahaemolyticus* and 2 *Escherichia coli* strains. We showed that the observed protective effect of *E. coli* was not correlated with a reduced host inflammatory response, nor with the release of biocidal molecules by protective bacteria, but rather with the presence of specific adhesion factors such as F pili that promote the emergence of probiotic bacteria in zebrafish larvae. Our study therefore provides new insights into the molecular events underlying the probiotic effect and constitutes a potentially high-throughput *in vivo* approach to the study of the molecular basis of pathogen exclusion in a relevant model of vertebrate oro-intestinal infection.

## Author Summary

The beneficial contribution of commensal bacteria to host health led to the concept that exogenous and non-pathogenic bacteria (probiotics) could be used to prevent infectious disease. However, the absence of relevant experimentally tractable *in vivo* models severely limits our understanding of the molecular processes behind probiotic effects, therefore hampering prophylactic and therapeutic use of probiotics against infections. Here we developed a protocol to raise microbe-free zebrafish larvae fed on microbe-free live food. We placed this microbiologically controlled model in contact with known pathogens and potential probiotics to investigate molecular events underlying pathogen exclusion by probiotic bacteria. We showed that *Edwardsiella ictaluri*, the causative agent of catfish enteric septicemia, causes rapid death of infected larvae following exposure via the natural immersion route. We used this mortality to screen potential probiotic bacteria able to extend zebrafish survival to *E. ictaluri* infection and thereby identified 3 protective strains. While host immune response modulation did not contribute to protection against *E. ictaluri* infection, comparison of protective and non-protective strains demonstrated a key role for their adhesion factors. Our *in vivo* approach constitutes a relevant new model of vertebrate oro-intestinal infection and provides new insight into molecular events underlying probiotic effects against incoming pathogens.

## Introduction

Non-pathogenic bacteria associated with animal mucosa contribute to host health and homeostasis by promoting key physiological functions and by providing protection against pathogen infections [1], [2], [3], [4]. This protection, induced upon stimulation of the host immune defenses or by direct bacteria-bacteria interactions, led to the concept that carefully chosen bacteria called probiotics could be introduced in natural host microbial communities to limit infection upon colonization by pathogens [5], [6], [7], [8].

Clinical evidence of probiotic efficacy in treatment of gastro-intestinal disorders and allergic symptoms triggered strong interest in the identification of biological mechanisms behind these beneficial effects [9]. Study of the protective role of probiotic bacteria during host-pathogen interactions, using comparative genomics and microbiologically controlled animal models such as gnotobiotic mice, rats, rabbits and pigs, has led to significant progress [10], [11], [12]. However, these mammal models are often complex and have low-throughput, while practical limits hamper identification of molecular processes behind probiotic effects, a prerequisite for prophylactic or therapeutic use of probiotics against infections [12], [13]. As an alternative to gnotobiotic mammal models, several invertebrates, including the fruit fly *Drosophila melanogaster* and the nematode worm *Caenorhabditis elegans*, have been used to study protection provided by commensal or probiotic bacteria against pathogens [14], [15]. New models are however needed to identify, select and evaluate factors involved in probiotic effects in a more relevant vertebrate context [4].

Recently, zebrafish (*Danio rerio*), a tropical freshwater cyprinid and successful model in vertebrate developmental biology, proved to be convenient for studying bacterial intestinal colonization and host-pathogen interactions [16]. Zebrafish have an innate immune system and develop adaptive immunity by the age of 6 weeks, and the development and physiology of its digestive tract are very similar to those of mammals [17], [18]. Moreover, germ-free zebrafish larvae are relatively easy to obtain and the small size and easy husbandry, combined with available genetic tools, make it particularly amenable to molecular analyses both from the host and bacterial point of view [19], [20], [21]. Thus far, more than twenty different bacteria have been used to infect zebrafish through various infection routes, providing valuable insight into host-pathogen interactions [16], [22], [23] and, more rarely, commensal/pathogen interactions within controlled intestinal microbial communities [24], [25], [26], [27]. Here we developed a new experimental approach for direct analysis of host and bacterial factors involved in protection provided by exogenous probiotic bacteria against pathogens. We raised axenic zebrafish larvae on axenic live food and screened a library of intestinal fish pathogens for virulent bacteria introduced in fish water and acquired by the natural route. We found that *Edwardsiella ictaluri*, the causative agent of catfish enteric septicemia, is responsible for rapid lethal infection. This simple read-out of premature death enabled us to carry out a secondary screen for Gram-positive and Gram-negative non-indigenous protective bacteria. We identified 3 strains robustly protecting zebrafish larvae out of 37 potential commensal probiotic bacteria. The analysis of immunological responses in larvae, which still only exhibit innate immunity, and of the outcomes of infection in pre-colonized zebrafish, demonstrated the protective role played by probiotic adhesion factors against *E. ictaluri*. Our *in vivo* model therefore provides a relevant and potentially high-throughput approach to oro-intestinal infection so as to elucidate key events underlying pathogen exclusion by probiotic bacteria.

## Results

### Raising axenic zebrafish using gnotobiotic live food

Studies of bacterial virulence in zebrafish have mainly been performed using conventional (i.e. non-axenic) larvae. To investigate the molecular bases of protection by non-indigenous probiotic bacteria against incoming pathogens in a microbiologically controlled zebrafish host, we produced germ-free zebrafish larvae by sterilizing freshly fertilized eggs using both antibiotic and chemical treatments, as previously described [20], [28]. These germ-free larvae hatched spontaneously between 3 and 4 days post-fertilization (dpf) and were first tentatively fed sterile autoclaved fish food powder. However, unless a large amount of powder having deleterious effects was used, as observed in [29], this procedure simply led to insufficient access of food particles to the mouth, likely due to poor elicitation of larval hunting behavior by non-moving food particles [30]. To circumvent this limitation and at the same time maintain adequate water quality, we fed newly hatched germ-free larvae every other day with live axenic *Tetrahymena thermophila*, a well studied ciliate advantageously substituting for natural zebrafish zooplankton prey [31] (Figure S1 in text S1). Standard body length [32], and growth rate of larvae fed with *T. thermophila* was similar in germ-free and conventionally raised larvae (data not shown). This enabled us to routinely raise germ-free larvae for up to 15 dpf at 28°C, as indicated by the absence of bacterial colony forming units (CFU) after plating and by negative 16S-based PCR analysis of water and homogenized larvae (data not shown). To raise axenic zebrafish beyond 15 dpf, we also fed larvae axenic *Artemia salina nauplii* from 10 dpf onwards, therefore extending the life span of axenic zebrafish up to at least 1 month, at which point they could efficiently feed on sterile food powder. However, raising zebrafish on *A. salina* nauplii was labor-intensive and less experimentally amenable to multiple analyses. Therefore, we used zebrafish larvae fed only *Tetrahymena* for the rest of this study.

### Identification of bacterial pathogens infecting zebrafish by the natural route

**Figure 1:**
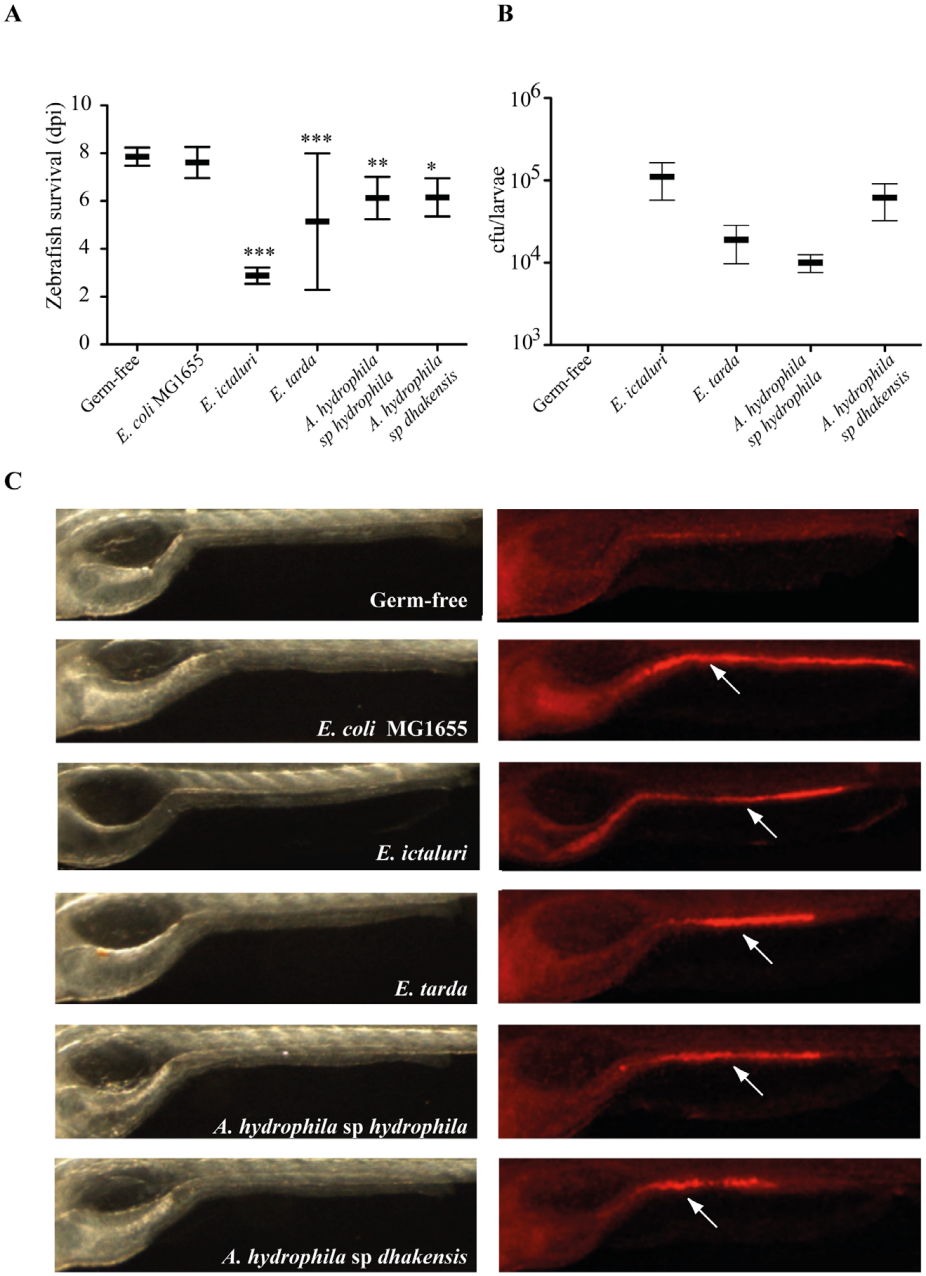
Life expectancy and colonization of zebrafish swimming larvae infected by different pathogenic bacteria. **A**. Life expectancy of axenic zebrafish larvae exposed by bath at 6 dpf to *E. coli*, *E. ictaluri* or other pathogenic bacteria. Mean survival is represented by a large hyphen. Standard deviations are also indicated. Asterisks indicate significant difference from non-infected population (*p<0.05, **p<0.01, ***p<0.001). **B**. Colonization of zebrafish larvae infected by different pathogenic bacteria. CFU counts of axenic zebrafish larvae exposed by bath at 6 dpf to *E. ictaluri* and other pathogenic bacteria. Mean and standard deviations are indicated. (n = 5). **C**. Colonization of zebrafish gut monitored at 9 dpf ( = 3 days post infection) by transmitted light microscopy (left) and whole-mount immunohistochemistry using a polyclonal antibody recognizing Gram-negative bacteria (right). Arrows indicate bacterial localization within the gut.

We reasoned that a relevant study of protective bacteria-bacteria interactions within the intestinal tract of axenic zebrafish larvae required prior identification of virulent intestinal pathogens able to infect their host via the natural route. We screened a total of 25 potential enteric fish pathogens, including 16 different species or subspecies and several different isolates of *Aeromonas*, *Vibrio*, *Edwardsiella*, *Listonella Photobacterium* and *Yersinia* (see Table S1 in text S1). At 6 dpf, axenic zebrafish larvae were brought in contact with each tested pathogen by immersion for 6 h in water containing bacteria adjusted to 2.10^8^ CFU/ml. After 6 h, larvae were washed and transferred to individual 24-well microtiter plate containing fresh autoclaved mineral water and incubated at 28°C under sterile conditions. Sterility of control germ-free larvae subjected to mock infection was monitored throughout the experiment by plating and 16S PCR analysis (data not shown). While contact with the non-pathogenic bacterium *Escherichia coli* MG1655 did not affect the viability of zebrafish larvae, daily monitoring of mortality upon contact with the tested pathogens enabled us to identify the channel catfish pathogen *E. ictaluri* as being highly pathogenic for zebrafish larvae, leading to high and reproducible mortality within 3 days after exposure (Figure 1A). We also observed that three other fish pathogens caused slightly premature mortality in zebrafish larvae: *Edwardsiella tarda* CIP 78.61, a human and fish pathogen, and two *Aeromonas* strains, *Aeromonas hydrophila* sp. *dhakensis* CIP 107500 and 1 out of 6 *Aeromonas hydrophyla* sp. *hydrophyla* strains (strain CIP 103561) [33], [34] (Figure 1A). Whole-mount immunohistochemistry using a polyclonal antibody recognizing various Gram-negative bacteria and CFU counts recovered from freshly euthanized homogenized infected larvae allowed us to confirm that these bacteria colonized the zebrafish gut (Figure 1BC).

### *E. ictaluri* infection induces peri-oral and intestinal lesions

**Figure 2:**
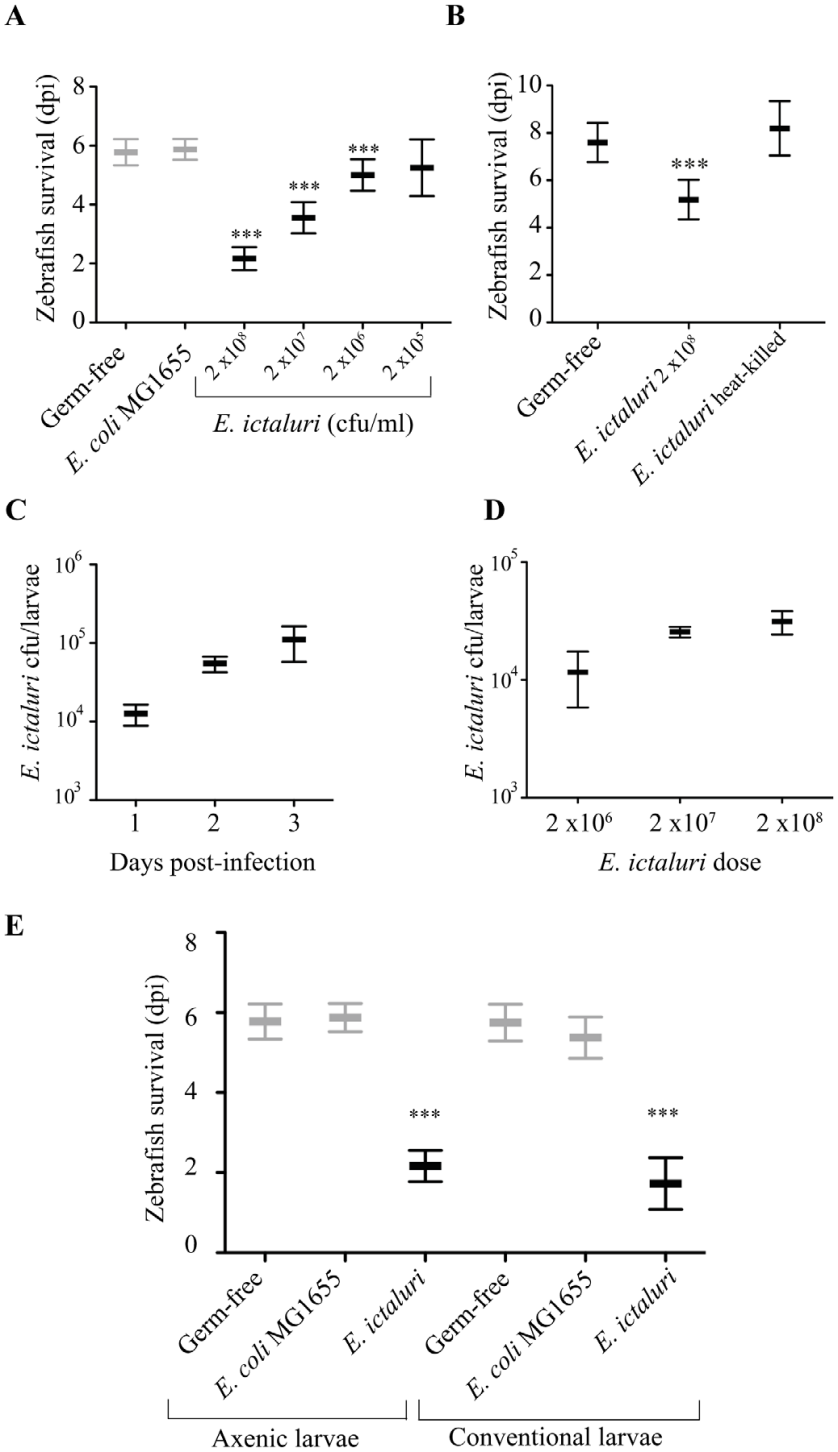
Characterization of zebrafish larva infection by *E. ictaluri*. 6 dpf germ free or conventional zebrafish larvae exposed to *E. coli* MG1655 or *E. ictaluri* by bath immersion were transferred after 6 h to clear autoclaved water. **A**. Influence of the amount of *E. ictaluri* in immersion bath on larvae mortality rate monitored daily and presented as in Figure 1. Control populations are shown in gray. **B**. *E. ictaluri* CFU number recovered from zebrafish larvae at different days post-infection (Mean ± SD; n = 5). **C**. *E. ictaluri* CFU number recovered from zebrafish larvae at 3 days post-infection when infected with different doses of *E. ictaluri* (Mean ± SD; n = 4). **D**. *E. ictaluri*-induced mortality of axenic and conventional zebrafish larvae monitored and presented as in panel A. **E**. *E. ictaluri*-induced mortality of axenic and conventional zebrafish larvae as monitored and presented as in panel A.

To characterize *E. ictaluri* infection, we first determined whether lethality towards germ-free zebrafish larvae was dose-dependent (Figure 2A), and not induced by dead heat-killed *E. ictaluri* (Figure 2B). The number of *E. ictaluri* bacteria recovered from freshly euthanized homogenized infected larvae increased between 1 and 3 days post infection (dpi), reaching levels of up to 4.8×10^5^ CFU/larva shortly before death (Figure 2C). Moreover, larvae infection with increasing dose of *E. ictaluri* also correlated with increased larvae colonization (Figure 2D). We then tested the impact of exposure to *E. ictaluri* on conventional larvae and found similar sensitivity to *E. ictaluri* infection in axenic and conventional larvae (mean survival reduced by 4 and 5 days, respectively; Figure 2E), suggesting that indigenous microbial communities developed at our facilities does not protect against *E. ictaluri* infection and that the absence of indigenous bacteria is not the main cause of the strong virulence of *E. ictaluri* in axenic zebrafish larvae.

**Figure 3:**
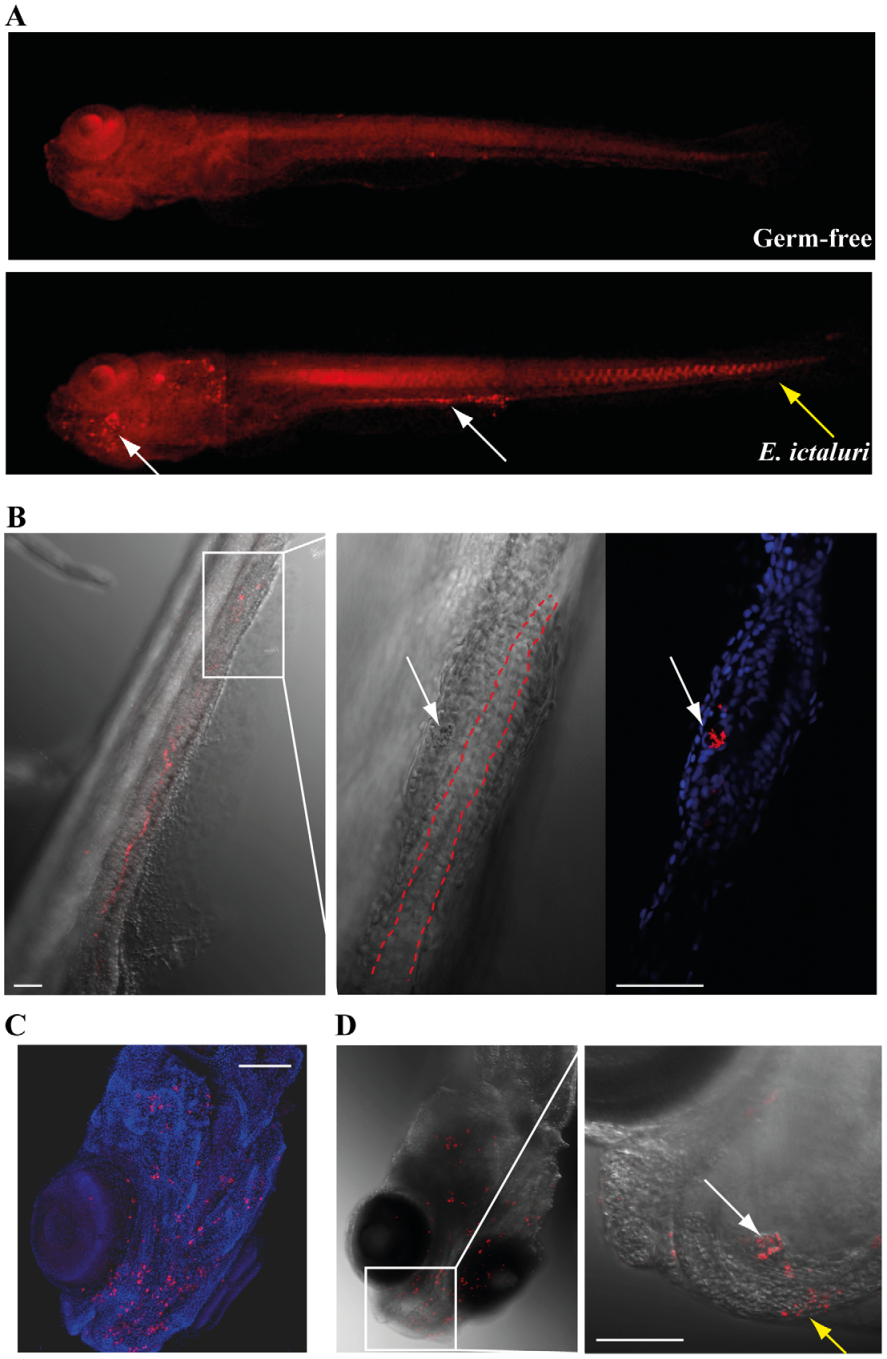
Localization of *E. ictaluri* in infected gnotobiotic zebrafish larvae. At 3 days post-infection ( = 9 dpf), germ-free zebrafish larvae exposed at 6 dpf to *E. ictaluri* were analyzed by whole-mount immunofluorescence. Germ-free 9 dpf zebrafish larvae were used as control. **A**. Localization of *E. ictaluri* in infected larvae. *E. ictaluri* cells (red) were detected with a stereomicroscope by immunofluorescence using a polyclonal antibody recognizing Gram-negative bacteria, including *E. ictaluri*. White arrows pinpoint *E. ictaluri* main infection sites on zebrafish head and gut. Yellow arrows pinpoint non-specific labeling. **B**. Details of *E. ictaluri* insertion in larval intestinal tissue. Clusters of *E. ictaluri* cells (shown by large white arrows) were observed by confocal microscopy outside the gut lumen, surrounded by zebrafish intestinal cells (nuclei stained in blue). Left panel: 10× objective, transmitted light and red (bacteria) fluorescence overlay; central panel: 40× objective, transmitted light, dashed red lines indicate gut lumen boundaries; right panel: 40× objective, red (bacteria) and blue (nuclei) fluorescence overlay. **C**. 10× objective. Confocal fluorescence picture of larval head infected by *E. ictaluri* (red). Zebrafish cell nuclei are shown in blue (DAPI staining). **D**. Analysis of larval rostrum by fluorescence and Nomarski optics. White arrow shows a bacterial abscess within the oral cavity, whereas the yellow arrow pinpoints *E. ictaluri* clusters co-localized with external skin lesions. White bars = 50 µm.

In its natural host, the primary route of entry of *E. ictaluri* is the intestine; however, several other potential infection routes have been reported, including olfactory sinus, gills and skin injuries [35], [36]. To determine *E. ictaluri* infection sites in axenic zebrafish larvae, the *E. ictaluri* localization in infected larvae was monitored over time by whole-mount immunofluorescence. Consistent with CFU counts, *E. ictaluri* immunofluorescence signals increased from 1 to 4 dpi until larval death and were mainly detected in the gut lumen and on the head underside (Figure 3A and data not shown). At 3 or 4 dpi, we sometimes observed small bacterial aggregates within the *lamina propria* of the distal intestine, indicating that a few bacteria had crossed the intestinal barrier (Figure 3B). In the series of samples from which this image has been obtained, we observed such breaches in gut epithelium in about half of the observed individuals, generally with a single event per fish; however this was not observed in all experiments. In contrast, *E. ictaluri* was consistently found around the mouth and/or inside multiple abscess-like lesions (42±20 (mean±SD) bacterial clusters of 10 µm or more in diameter) located on skin surfaces from the jaw to the gill area, or within the oral cavity (Figure 3CD, Video S1 and Video S1 caption in text S1).

Altogether, these results showed that *E. ictaluri* entry into zebrafish larvae upon exposure by immersion led to both abundant abscesses in the peri-oral area and intestinal colonization, with occasional crossing of the intestinal barrier.

### Immune responses induced upon *E. ictaluri* infection

**Figure 4:**
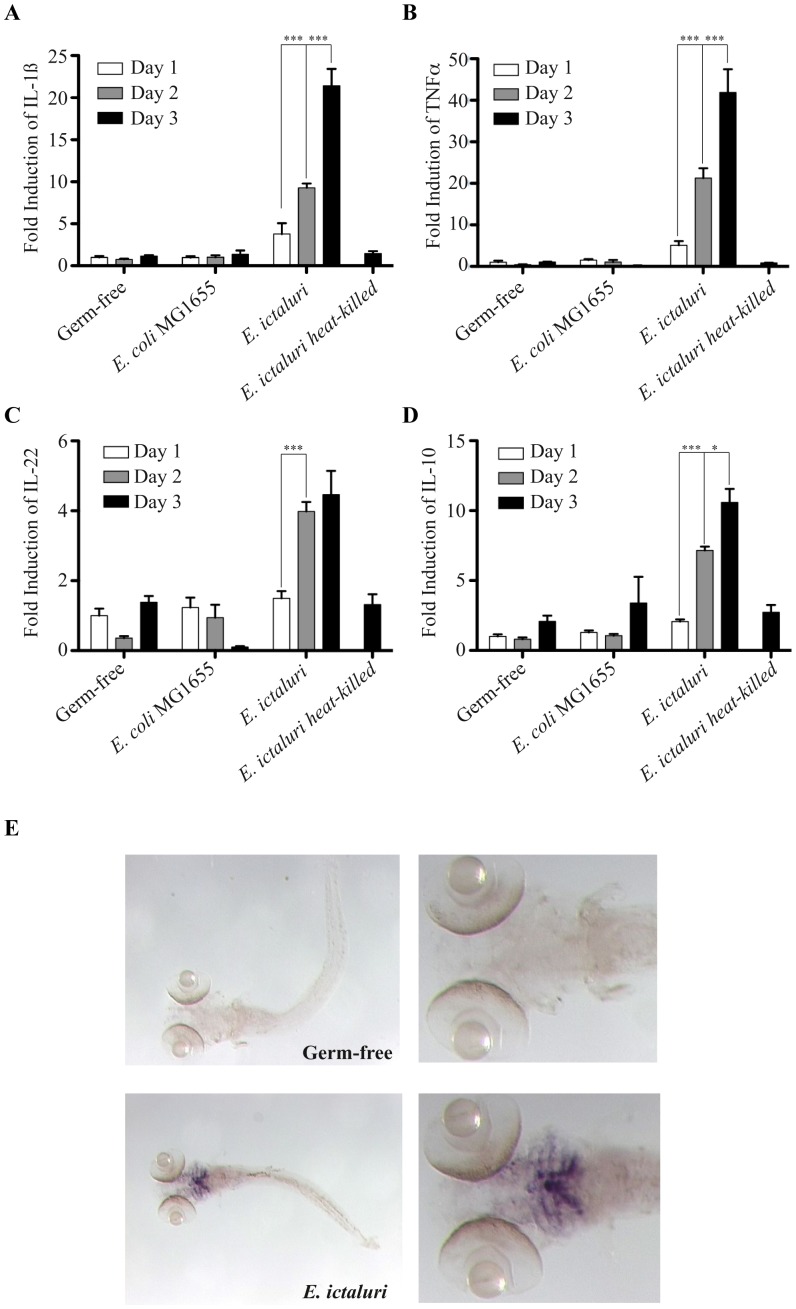
Characterization of the gnotobiotic zebrafish larva immune response to *E. ictaluri* infection. Kinetics of inflammation marker expression in zebrafish larvae. qRT-PCR was performed using primers specific to *il1b* (**A**), *tnfa* (**B**), *il22* (**C**) and *il10* (**D**) (inflammation markers) on RNA extracted from pools of germ-free zebrafish larvae or larvae exposed to *E. coli* MG1655 (control) or *E. ictaluri* at 1, 2, and 3 days post-infection or heat-killed 3 dpi *E. ictaluri*. Levels were standardized to levels of uninfected axenic fish, presented results are mean±SEMof three biological replicates. Asterisks indicate significant difference determined by two-way ANOVA with Bonferroni correction (*p<0.05, **p<0.01, ***p<0.001). **E**. Localization of *il1b* expression in zebrafish larvae performed by *in situ* hybridization on whole-mounted zebrafish larvae treated at 3 dpi.

To study the impact of bacterial infection on host immunological responses, we monitored mRNA levels of genes encoding inflammation markers TNFα, IL-1β, IL-22 and IL-10, including pro- and anti-inflammatory cytokines, in axenic and infected zebrafish larvae at 1, 2 and 3 dpi. Using quantitative RT-PCR, we observed that, while the levels of transcripts for all four cytokines remained low in axenic zebrafish larvae and in larvae exposed to the non-pathogenic bacteria *E. coli* MG1655, they were higher and increased significantly over time in larvae infected by *E. ictaluri* (Figure 4A–D). This increase required live bacteria and not only their epitopes, since incubation with heat-killed *E. ictaluri* did not induce inflammation (Figure 4A–D) nor did it reduce lifespan of larvae (data not shown). A similar analysis performed with larvae infected by the 3 other milder pathogens identified in our screen (*E. tarda*, *A. hydrophila* sp *dhakensis*, *A. hydrophila* sp *hydrophila*) also revealed that they marginally induced cytokine transcripts (Figure S2 in text S1). Consistent with the localization of *E. ictaluri* in infected larvae, whole-mount *in situ* hybridization at 3 dpi revealed clusters of *il1b*-expressing cells in the head region, especially in the gill arches and next to localized skin lesions (Figure 4E).

The localization of neutrophils was also monitored over time during colonization using *mpx:gfp* transgenic zebrafish larvae [37]. At 3 or 4 dpi, while neutrophils were found distributed throughout the body in germ-free zebrafish and in larvae colonized by control strain *E. coli* MG1655, larvae infected with *E. ictaluri* displayed strong neutrophil recruitment to the peri-oral region (Figure S3 in text S1). Unexpectedly, enterocytes were also seen to express GFP in *E. ictaluri*-infected fish, but this did not hamper identification of neutrophils. In contrast, mild pathogens did not induce significant neutrophil recruitment in the head and gut (Figure S3 in text S1). These observations were confirmed in wild-type larvae stained with Sudan black, a dye that specifically labels neutrophil granules (data not shown) [38].

### Identification of bacteria protecting against *E. ictaluri* infection in pre-colonized zebrafish larvae

**Figure 5:**
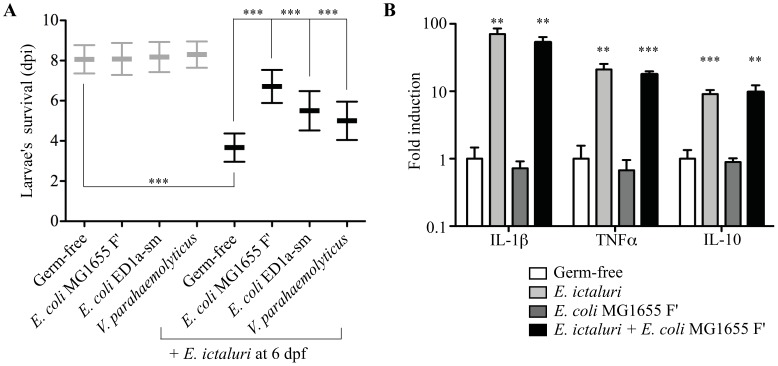
Protective effect of selected strains against gnotobiotic zebrafish larva infection by *E. ictaluri*. **A**. Four dpf-old freshly hatched axenic zebrafish larvae were kept germ-free or incubated with selected protective bacterial strains for 2 days prior to exposure to *E. ictaluri* at 6 dpf. Mortality was monitored daily after *E. ictaluri* infection. Control non-infected larvae are shown in gray. Asterisks indicate significant difference from *E. ictaluri*-infected population (***p<0.001). **B**. Inflammation marker expression in pretreated *E. ictaluri*-infected larvae. qRT-PCR was performed on 5 individual larvae per group using primers specific to *il1b*, *tnfa* and *il10* on RNA extracted at 3 dpi ( = 9 dpf) from germ-free or *E. coli* MG6155 F′ pre-colonized zebrafish larvae exposed to *E. ictaluri* at 3 days post infection. Results were normalized to mean expression in germ-free animals; mean ± SEM. Asterisk indicates significant difference between *E. ictaluri*-infected and uninfected populations (**p<0.01, ***p<0.001).

We hypothesized that larval mortality following *E. ictaluri* infection could be used as a phenotypic read-out to reveal protective effects provided by known probiotic bacteria. For this, we pre-colonized freshly hatched (4 dpf; see Figure S1 in text S1) axenic zebrafish larvae with 37 commensal or probiotic Gram-positive and Gram-negative bacteria often used as probiotic strains in the food industry and/or aquaculture, including several *E. coli*, *Lactobacillus* spp., *Pediococcus* spp., *Pseudomonas, Phaeobacter, Aeromonas and Vibrio* strains (see Table S2 in text S1). These pre-colonized larvae were then infected at 6 dpf with *E. ictaluri* and we compared their mortality rate with axenic or non-infected larvae colonized only by a probiotic bacterium. This screen showed that pre-incubation with *V. parahaemolyticus*, *E. coli* ED1a-sm and *E. coli* MG1655 F′, a strongly adherent and biofilm-forming commensal, provided a significant increase in survival upon *E. ictaluri* infection (Figure 5A and Table 1).

**Table 1:** Strains and plasmids used in this study.

Name	Genotype or main characteristics	Antibiotic resistance	Reference
*E. coli strains*			
MG1655	*E. coli* K-12 derivative		[68], [69]
MG1655 F×	K-12 MG1655 *attB*::*gfp*-*bla*_(F′*tet* Δ*traD::apra* Δ*tetR::zeo tetA*::Tn*luxCDABE*-Km)	Amp^R^, Apra^R^, Zeo^R^, Km^R^	Lab collection
MG1655*km*PcL*fim_gfp*	*fimAICDFGH* operon with its own RBS sequence placed under the control of the *km*PcLrbs cassette λP_R_ promoter in MG1655*gfp*. GFP+	Km^R^, Amp^R^	[70]
MG1655Δ*fim_gfp*	*fimAICDFGH::cat* in MG1655_*gfp*, GFP+	Cm^R^, Amp^R^	[70]
MG1655 λatt gfp ΔompR234_*malA*::Km	Strain constitutively expressing curli, GFP+	Km^R^, Amp^R^	[71]
MG1655*km*PcL*flu_gfp*	*flu* (*ag43*) with its own RBS sequence placed under the control of the *km*PcLrbs cassette λP_R_ promoter inMG1655_*gfp*.	Km^R^, Amp^R^	[71]
*E. coli* ED1a	WT. Human feces from healthy man (France).		[72]
*E. coli* ED1a-sm	Spontaneous streptomycin-resistant mutant of ED1a	Strep^R^	This study
*E. coli* ED1a-sm F′	ED1a-sm F′*tet*	Tet^R^	This study
*Other strains*			
*E. ictaluri*	*Edwardsiella ictaluri* CIP 81.96 (WT)		CRBIP
*E. tarda*	*Edwardsiella tarda* CIP 78.61 (WT)		CRBIP
*A. hydrophila* sp. *hydrophila*	*Aeromonas hydrophila* sp. *hydrophila* CIP 103561 (WT)		CRBIP
*A. hydrophila* sp. *dhakensis*	*Aeromonas hydrophila* sp. *dhakensis* CIP 107500(WT)^−^		CRBIP
*Vibrio parahaemolyticus*	CIP 109835		CRBIP

**CRBIP:** *Centre de Resources Biologiques de l'Institut Pasteur,*

### Monitoring of bacterial and host factors for probiotic effects against *E. ictaluri* infection

**Figure 6:**
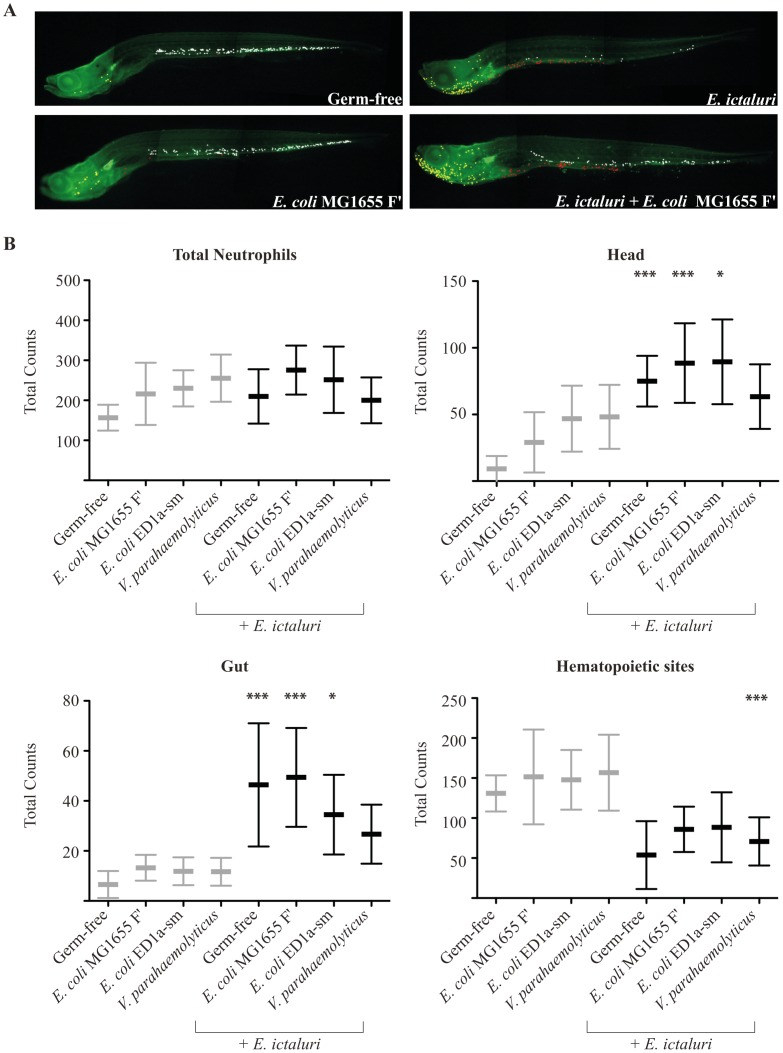
Comparative analysis of neutrophil distribution in pretreated larvae infected or not by *E. ictaluri*. The distribution of neutrophils after *E. ictaluri* infection was examined at 9 dpf in *mpx*:GFP larvae stained with an anti-GFP antibody (n = 5 to 10 per condition) (**A**) Representative pictures from which neutrophils were manually counted are shown. Total neutrophils consist of all visible neutrophils except those in the kidney, which is too deep and too dense for cells to be accurately counted. Among these, three subpopulations were counted: head (yellow dots), hematopoietic sites in the trunk and tail (white dots) and gut (red dots). (**B**). Neutrophil counts; statistical significance was calculated between the corresponding pretreated larvae infected and non-infected by *E. ictaluri*. (*p<0.05, **p<0.01, ***p<0.001).

To investigate possible direct interactions between *E. ictaluri* and the three identified protective strains, we first showed that *in vitro* exposure to *E. coli* MG1655 F′ or *E. coli* ED1a-sm bacterial-free supernatants did not impair *E. ictaluri* growth nor biofilm formation in microtiter plate assays (Figure S4AB in text S1). By contrast, *V. parahaemolyticus* supernatant slightly reduced *E. ictaluri* growth of (Figure S4B in text S1). Consistently, while broth co-cultures with *E. coli* MG1655 F′ or *E. coli* ED1a-sm did not reduce *E. ictaluri* cfu count compared *E. coli* MG1655 strain, co-culture with *V. parahaemolyticus* reduced *E. ictaluri* growth rate, suggesting a potentially distinct protection mechanism for *V. parahaemolyticus* (Figure S4C in text S1). We then compared transcription levels of *il1b*, *tnfa* and *il10* in larvae pre-incubated with the most protective strain (*E. coli* MG1655 F′), infected or not by *E. ictaluri*. Whereas no inflammation could be detected in larvae colonized only by *E. coli* MG1655 F′, all markers were still strongly induced upon *E. ictaluri* infection of pre-incubated larvae despite observed protective effects (Figure 5B). However, pre-colonization with *V. parahaemolyticus* induced some cytokine gene expression, suggesting potential differences in the mechanisms of action of the various protective strains identified (data not shown). This difference was also observed when studying the distribution of a neutrophil population of *E. ictaluri*-challenged larvae when pre-colonized or not by probiotic strains (Figure 6A). Total counts of visible neutrophils did not significantly change under the different conditions tested, but were lower in germ-free animals. However, we found a significant redistribution of neutrophils to the head and gut at the expense of hematopoietic tissues in germ-free larvae infected by *E. ictaluri* (Figure 6B and S5 in text S1). Similar redistribution was also found in larvae pre-colonized by *E. coli* MG1655 F′ or *E. coli* ED1a-sm and infected by *E. ictaluri* (Figure 6B). In contrast, larvae pretreated with *V. parahaemolyticus* and infected with *E. ictaluri* display neutrophil distribution similar to that of uninfected larvae, except for a reduction in hematopoietic tissues (Figure 6B). These results further suggested that mechanisms of protection against *E. ictaluri* infection differ between protective strains.

To specifically quantify infection with *E. ictaluri*, we developed a qPCR-based assay from DNA extracted from entire larvae. This assay did not show significant variation in the level of *E. ictaluri* colonization in germ-free vs MG1655 or MG1655 F′ precolonized larvae (Table S4). The distribution of bacteria in pre-colonized larvae challenged with *E. ictaluri* was assessed by whole-mount immunohistochemistry using a polyclonal antibody recognizing various Gram-negative bacteria. Although this antibody does not discriminate between protective bacteria and pathogens, abscesses were consistently observed in the peri-oral region, suggesting that protective bacteria did not impair infection of the head by *E. ictaluri*. By contrast, while no crossing of the gut barrier by *E. ictaluri* was observed when larvae were pretreated with the protective strains, we could not reach definitive conclusions regarding gut infection.

### Role of bacterial adhesion in zebrafish protection against *E. ictaluri* infection

**Figure 7:**
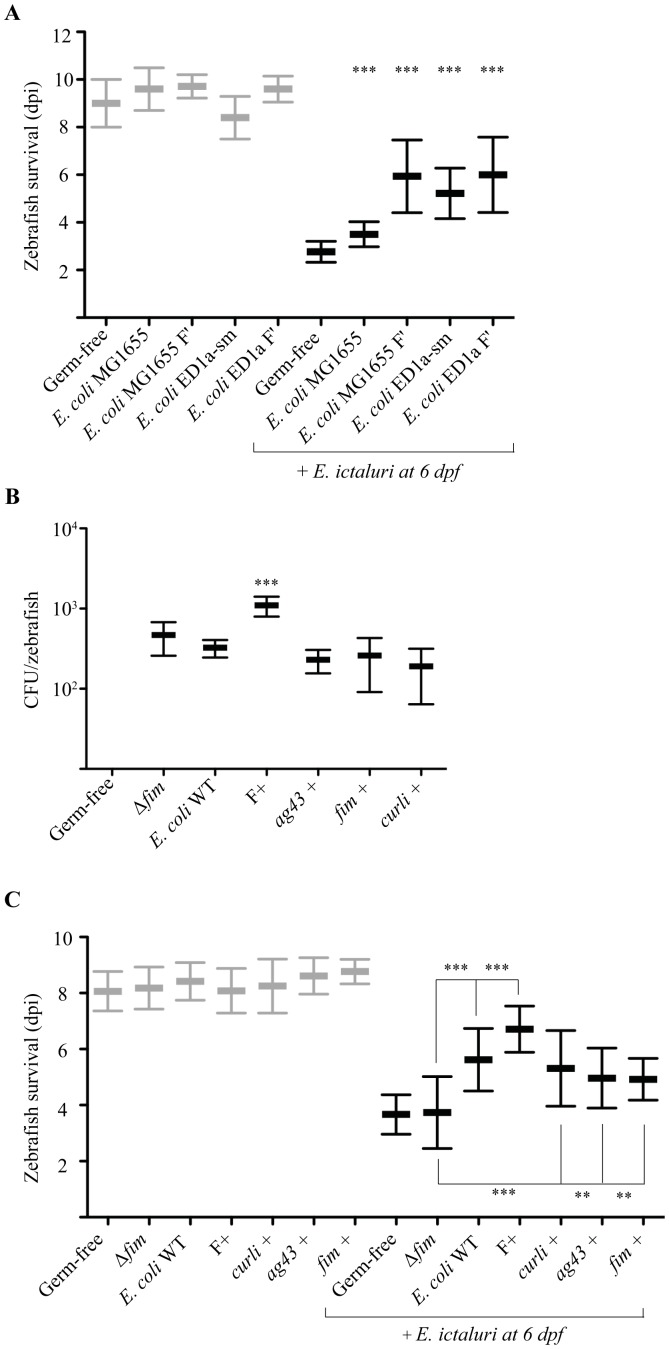
Impact of *E. coli* adhesive properties on its protective effect in *E. ictaluri*-infected zebrafish larvae. **A**. Effect of introduction of conjugative F plasmid in other *E. coli*. Four dpf-old freshly hatched larvae were kept germ-free or incubated with *E. coli* MG1655 or *E. coli* ED1a-sm with and without the F plasmid for 2 days prior to exposure to *E. ictaluri* at 6 dpf. Mortality was monitored daily after *E. ictaluri* infection. Controls are shown in gray (***p<0.001). **B**. Quantification of *E. coli* expressing different adhesion factors associated with gnotobiotic zebrafish larvae at 9 dpf. Means and standard deviations of the number of CFU recovered from larvae are reported (n = 4; ***p<0.001). Abbreviations used: F+ for F plasmid; *ag43*+ for antigen 43; *fim* for type 1 fimbriae; WT for wild type. **C**. Mortality rate of larvae pretreated with *E. coli* derivative strains constitutively expressing different adhesion factors and infected or not (control –gray-) with *E. ictaluri* (***p<0.001).

Our results indicated that reduced *E. ictaluri* virulence by *E. coli* MG1655 F′ did not result from direct toxicity, nor from a change in the zebrafish inflammatory response. *E. coli* MG1655 F′ is a highly adherent derivative of MG1655 that carries the F conjugative plasmid and expresses F pili involved in conjugation and biofilm formation [39]. Since zebrafish larvae pre-incubated with wild type *E. coli* MG1655 only poorly protected against *E. ictaluri* infection (Figure 7A), this suggested that the protective effect of by *E. coli* MG1655 F′ might stem from changes induced by the F plasmid. Moreover, we showed that MG1655 F′ was able to colonize zebrafish larvae better than wild-type MG1655 (Figure 7B), indicating that protection of *E. ictaluri* infected larvae was correlated with the ability of MG1655 to colonize zebrafish, both in axenic and conventional larvae (Figure S6 in text S1) To further elucidate the mechanism of this protection in MG1655 background, we used an F plasmid carrying a conjugation-deficient *traD* mutant that still produces the F pili adhesin (Table 1). We found that this mutant continued to increase the life expectancy of *E. ictaluri*-challenged zebrafish larvae, indicating that the protective function is independent of conjugation events (Figure 7A). In addition, introduction of an F plasmid in the protective *E. coli* ED1a-sm did not significantly increase protection of pre-incubated larvae against *E. ictaluri* infection (p = 0.07), potentially due to the already strong ability of ED1a-sm to colonize zebrafish larvae compared to *E. coli* MG1655 (Figure 7A and Table S3 in text S1).

*E. coli* MG1655 wild type has several adhesion factors shown to increase bacterial attachment to various surfaces, including type 1 fimbriae, curli and antigen 43 [40]–[42]. To determine whether bacterial adhesion could be a key molecular determinant in MG1655 F′ protection against *E. ictaluri*, we tested whether increased bacterial adhesion correlated with increased protection. For this, we pre-incubated axenic larvae with *E. coli* derivatives constitutively expressing different adhesins previously shown to increase bacterial adhesion to various surfaces, such as antigen 43 (Ag43), type 1 fimbriae and curli [40]–[42]. Monitoring of bacterial colonization at 9 dpf in homogenized larvae pre-incubated with these MG1655 derivatives showed that these strains did not show increased ability to colonize axenic larvae when compared to the MG1655 wild type (Figure 7B). Consistently, these strains did not further delay *E. ictaluri* infection when compared to the MG1655 wild type (Figure 7C). However, deletion of type 1 fimbriae operon genes showed that *E. coli* MG1655 Δ*fim* was no longer able to protect against *E. ictaluri* infection (Figure 7C). Type 1 fimbriae were involved in adhesion to intestinal and epithelial cells in different *E. coli* strains such as K1-type *E. coli* [43], avian pathogenic *E. coli* [44], enteroaggregative LF82 [45] and the probiotic Nissle strain [46]. Our results therefore suggested that type 1 fimbriae, and potentially other *E. coli* adhesins, could contribute to zebrafish tissue adhesion, reaching its maximum under wild-type conditions, since overexpression did not lead to further protection and colonization (Figure 7BC). Altogether, these data indicate that *E. coli* MG1655 F′ adhesion capacity provided by the F-plasmid and to a lesser extent type 1 fimbriae, is involved in protection against *E. ictaluri* infection.

## Discussion

Over a century ago, Elie Metchnikoff postulated the existence of the probiotic effect [47]; however, few of its actual mechanisms were experimentally demonstrated *in vivo*, thus severely limiting the scope of applications of probiotics in alternative anti-infectious strategies [12], [47]. Here we developed a controlled model of axenic vertebrate colonization to study host and bacterial aspects of probiotic-based protection against bacterial pathogens acquired by a natural route of infection.

We first circumvented current limitations of existing procedures to raise axenic zebrafish and we used this new protocol to study the impact of gut microbial communities on animal health and development [20]. While use of sterile fish food powder or even the absence of feeding [28], [48], [49] leads to rapid epidermal degeneration followed by premature death [27], we show here that feeding axenic live food to zebrafish larvae enabled us to routinely raise gnotobiotic larvae for over a month. We hypothesize that the natural motility of *Tetrahymena* cells and *Artemia* naupli enables young larvae to more easily feed while food continuously remains in suspension. Moreover, multiplication of *Tetrahymena* cells on waste reduces food-based soiling of water in microtiter plate wells, thereby diminishing the requirement for frequent food supply. Finally, our procedure mimics natural feeding behavior and reduces the impact of nutritional parameters on fish development and outcome of intestinal microbe-host interactions. Although we did not systematically raise older larvae, 1-month-old axenic zebrafish appeared morphologically healthy, suggesting that axenic zebrafish can be raised over a longer period. This new and reproducible procedure therefore opens up the prospect of studying the impact of bacteria on zebrafish gut anatomy and physiology from larval to the adult stage.

Zebrafish constitute an increasingly popular model for analyzing bacteria-host interactions and bacterial pathogenicity *in vivo* [16], [17], [19], [50], [51], and many studies have used inoculation infection procedures such as intramuscular or intraperitoneal injection in adults or in recently hatched larvae [16]. However, in addition to some viral pathogens [52], [53], to our knowledge, the only models of non-adult zebrafish infection by immersion used the pathogenic bacteria *E. tarda* and *Flavobacterium columnare* [54], [55], [56]. Those studies used 24 h immature embryos which did not yet have an open digestive system, and revealed modest infection efficiency and, when assessed, high variability. Hence, a critical advantage of our approach over these other models of infection by immersion is the high rate of disease incidence, allowing the use of manageable number of animals to reach statistical significance.

Here, axenic zebrafish larvae were placed in contact with a large panel of pathogens and probiotics by mere immersion in bacteria-containing water. This colonization procedure led us to identify several pathogenic bacteria, including *E. ictaluri*, a virulent strain rapidly deadly toward axenic and conventional zebrafish larvae. *E. ictaluri* is an enterobacterium identified as the causative agent of enteric septicemia in channel catfish (*Ictalurus punctatus*); it causes substantial economic losses, affecting most fish farms and ponds in the United States [57]. While epizootic diseases associated with acute septicemia caused by *E. ictaluri* have been observed only in channel catfish, this bacterium was also recovered from several other fish, including the madtom *Noturus gyrinus*, the Vietnamese freshwater catfish *Pangasius hypophtalmus*, the walking catfish *Clarias batrachus*, the green knifefish *Eigemannia virescens* and the Bengal danio *Danio devario*, and was also shown to be highly pathogenic when injected into adult zebrafish [58]. The relevance of our zebrafish model is further underlined by the observation that the *E. ictaluri* 93–146 virulent catfish isolate used by Karsi *et al.* [59] also led to high zebrafish mortality in our model, while its non-virulent derivative 65ST turned out to be non-pathogenic (data not shown) [60].

Fish pathogens generally enter into their host through the gills, skin and gastrointestinal tract, and the integrity of these physical and immunological barriers determines the outcome of host-pathogen interactions [61]. Although the natural infection route of *E. ictaluri* in its natural hosts is poorly characterized [35], [36], [59], we observed the early appearance of head cutaneous ulcers in the ventral head and lesions of intestinal tissue at later stages of infection (3 to 4 days post-infection), prior to larval death. While we can only but speculate about causes of fish mortality, use of the neutrophil reporter zebrafish line *mpx::gfp* suggests one possible scenario. Indeed, at an advanced stage of infection, neutrophils have relocalized from hematopoietic tissue to head and gut sites of infection. However, data obtained during early infection also suggested that neutrophils migrate first to the head only, therefore potentially leaving the gut with a transient weakening of the immune barrier against *E. ictaluri* intrusion (data not shown). This hypothesis is consistent with predominant expression of *il1b* in the head, as seen by *in situ* hybridization.

In addition to mortality induced upon *E. ictaluri* infection, we found that milder fish pathogens identified in our study–*E. tarda*, *A. hydrophyla* sp. *hydrophyla*, and *A. hydrophila* sp. *dhakensis*–also had an immunological impact upon infection of zebrafish larvae. Although this relatively small number of identified pathogens among previously described fish pathogens could be a consequence of host-specificity, we cannot exclude the possibility that some of the tested pathogens induced milder effects undetected in our stringent phenotypic screen.

We used *E. ictaluri* lethal infection as a phenotypic read-out to investigate potential protection provided by commensal or probiotic bacteria. Several mechanisms have been proposed to explain beneficial probiotic effects, including stimulation of the host immune system, production of antimicrobial compounds or competition for the attachment site or nutrients [5]. However, few of these mechanisms were actually shown to occur *in vivo* [12]. Here we show that pre-colonization of axenic zebrafish by *E. coli* MG1655 F′, *E. coli* ED1a-sm and *V. parahaemolyticus* protected the host against *E. ictaluri*. While many human probiotics, including several *Lactobacilli*, were tested in our study, none showed significant protective effect against *E. ictaluri* infection, possibly due to the aerobic nature of microbial communities hosted by zebrafish larvae. Whereas no *in vitro* or *in vivo* growth or colonization inhibition of *E. ictaluri* by the protective *E. coli* strains could be detected, we showed that *V. parahaemolyticus* impaired growth in broth co-cultures suggesting a distinct protection mechanism potentially involving contact-dependent toxicity or interference against *E. ictaluri*. Furthermore, monitoring of cytokine gene expression in infected zebrafish larvae pre-incubated or not with protective strains showed no attenuation of the zebrafish inflammatory response induced upon *E. ictaluri* infection. Although we may have missed modulation of other markers, the tested genes (*tnfa, il1b, il10, il22*) represent major actors in inflammatory responses and cover a variety of functions. In mammals, TNFα and IL1β constitute classical pro-inflammatory cytokines, known to activate leukocytes and endothelial cells among other cell types. IL-10 is also a well-known inflammation marker, but with pleiotropic anti-inflammatory functions. IL-22 is a more recently discovered cytokine that plays a protective role in bacterial infections by signaling to non-immune cells only, such as epithelial cells of the gastrointestinal tract and skin [62]. In contrast, enhanced colonization and the life expectancy of infected larvae in the presence of the biofilm-forming *E. coli* MG1655 F′ strain suggest that strong adhesion promoted by F pili could lead to *E. ictaluri* exclusion. While this exclusion could be due to direct competition upon *E. coli* MG1655 F′ adhesion to zebrafish intestinal tissues, other mechanisms could contribute to the observed protection effect, including alteration of tissue architecture, or modification of *E. ictaluri* behavior in pre-colonized larvae.

Hence, this suggests that the non-pathogenic *E. coli* MG1655 F′ strain or engineered derivatives could be used as potential probiotic strains against *E. ictaluri* or its closely related human pathogen *E. tarda* [63], [64]. Introduction of the F-plasmid into the already strong zebrafish colonizer ED1a-sm led to only a slight increase in protection of pre-incubated larvae, suggesting secondary mechanisms for a probiotic effect in ED1a-sm. In support of this hypothesis, we observed that the absence of type 1 fimbriae impaired the *E. coli* MG1655 ability to protect zebrafish larvae without affecting colonization. Furthermore, mechanisms by which *E. coli* MG1655 F′ protects zebrafish upon *E. ictaluri* infection might be different from those of *V. parahaemolyticus*, as evidenced by a differential inflammatory response and redistribution of neutrophils upon infection.

In conclusion, we have developed a new and potentially high-throughput approach to investigating competitive exclusion and protection against pathogens in microbiologically controlled zebrafish. Direct experimental analysis of a protective effect in a genetically tractable vertebrate model organism should prove useful when studying host-pathogen interactions. It will contribute to improvising the molecular definition of probiotic effects and their use in preventive and curative treatments against pathogens.

## Materials and Methods

### Ethics statement

All animal experiments described in the present study were conducted at the Institut Pasteur according to European Union guidelines for handling of laboratory animals (http://ec.europa.eu/environment/chemicals/lab_animals/home_en.htm) and were approved by the Institut Pasteur Animal Care and Use Committee and the Direction Sanitaire et Vétérinaire de Paris under permit #A-75-1061.

### Bacterial strains, plasmids and growth conditions

Bacterial strains and plasmids used in this study are listed in Table 1 and Tables S1 and S2 in text S1. *E. ictaluri* cells were grown in brain-heart infusion medium at 30°C; *E. tarda* and *Aeromonas* strains were grown in tryptic soy broth (TSB) supplemented with 0.25% glucose at 30°C. *Lactobacillus* strains were grown at 30°C in MRS medium. All other strains were grown in LB (lysogeny broth) at 37°C unless indicated otherwise. When required, antibiotics were added to the medium at the following concentrations: ampicillin (Amp, 100 µg/ml), chloramphenicol (Cm, 25 µg/ml), kanamycin (Km, 50 µg/ml), streptomycin (Sm, 100 µg/ml), tetracycline (Tc, 7.5 µg/ml). Proper formation of isolated *E. ictaluri* CIP 81.96 colonies on plates was achieved by supplementing BHI with 100 U/ml of bovine liver catalase (SIGMA C1345).

### Handling of zebrafish

Wild-type AB purchased from the Zebrafish International Resource Center (Eugene, OR, USA), or their F1 offspring, and *nacre* (melanocyte-deficient) mutants were raised in our facility. Eggs were obtained by marble-induced spawning and bleached according to protocols described in The Zebrafish Book [65]. After spawning, all procedures were performed in a laminar microbiological cabinet and with single-use disposable plastic ware. Fish were kept in vented cap culture flasks or 24-well microtiter plates in autoclaved mineral water (Volvic) at 28°C. Fish were fed every two days with axenic *T. thermophila*. For experiments running over 18 days, larvae were fed from day 10 onwards with axenic *Artemia salina*. To avoid waste accumulation and oxygen limitation, we renewed at least half the volume of water every two days to keep young zebrafish healthy.

### Sterilization of zebrafish eggs

Colonization of zebrafish mucosae by bacteria present on the surface of the chorion occurs rapidly after hatching [28]. To prevent it, freshly fertilized zebrafish eggs were sterilized by separating eggs into 50 ml Falcon tubes (100 eggs per tube) and washed 3 times in 50 ml of water (3 min at room temperature under smooth agitation). Afterwards, eggs were treated with a mixture of antibiotics (500 µl of penicillin G: streptomycin (10,000 U/ml: 10 mg/ml GIBCO #P4333), 200 µl of filtered kanamycin sulfate (25 mg/ml) SERVA Electrophoresis #26899) and antifungal drug (50 µl of amphotericin B solution Sigma-Aldrich (250 µg/ml) #A2942) for 4.5 h under agitation at room temperature. Then they were washed 3 times as described above. Next, they were bleached (0.003%) for 15 min, resuspending the eggs every 3 min. Eggs were washed again 3 times in water and transferred to Petri dishes to be distributed into 25 cm^3^ culture flasks with vented caps containing 10 mL of water (15 eggs/flask). We monitored sterility at several points during the experiment by spotting 50 µL from each flask either on tryptic soy medium agar plates supplemented with glucose or on YPD plates, all incubated at 28°C under aerobic conditions. Plates were left for at least 3 days to allow slow-growing organisms to multiply. If a particular flask was contaminated, those fish were removed from the experiment. The absence of any contamination by microorganisms in the fish larvae was further confirmed by PCR using primers specific for the chromosomal 16S region.

### Procedure for raising axenic zebrafish

*T. thermophila*. (*i*) Stock. A gnotobiotic line of *T. thermophila* was maintained at room temperature in 20 ml of PPYE (0.25% proteose peptone BD Bact#211684, 0.25% yeast extract BD Bacto# 212750) supplemented with 200 µl of penicillin G (10 unit/ml) and streptomycin (10 µg/ml). Medium was inoculated with 100 µl of the preceding *Tetrahymena* stock. After one week of growth, samples were taken, tested for sterility on TSB-glucose and YPD plates and restocked again. (*ii*) Growth. *T. thermophila* were incubated at 30°C under agitation in MYE (1% milk powder, 1% yeast extract) inoculated directly from stock at a 1∶50 ratio. After 24 h of growth, *Tetrahymena* were transferred to Falcon tubes and washed (1500 rpm, 10 min at 19°C) 3 times in 50 ml of autoclaved Volvic water. Finally, *Tetrahymena* were resuspended, transferred to culture flasks with vented caps and conserved for 4–5 days.

*Artemia salina*. *(i)* Rehydration. 5 ml of dehydrated cysts, conserved at 4°, were placed in a microfermentor (provided with sterile air input as well as a medium entry and exit) containing 40 ml of sterile PBS and left with ventilation for 1 h at 28°C. They were collected in a final volume of 14 ml of PBS. *(ii)* Decapsulation. 14 ml of rehydrated cysts were placed in a microfermentor and 16 ml of bleach (9.6%) were added. With abundant ventilation, in several minutes, the cysts turned from brown to orange. At this point, cysts were collected with a 0.18 mm large sieve (Hobby Aquaristik #21620) and rinsed with water until bleach was eliminated. *(iii)* Hatching. One ml of cysts was placed in a microfermentor containing 35 ml of filtered sea water (1 L of pyrolyzed water complemented with 20 g of “Instant Ocean” salts, 400 µl of sterile 1 M CaCl_2_ and 20 µL of sterile 1 M NaHCO_3_) and 20 µL of sterile NaHCO_3_ (1 M), then incubated for 24 to 48 h at 28°C with constant ventilation and continuous flow of sea water (125 ml per 24 h) to replace evaporated medium. *Artemia* were recovered with the sieve and abundantly washed with sterile PBS. They were finally resuspended in 30 ml of PBS, diluted 1∶10, counted and given to larvae (100 artemia/larvae).

### Pathogen infection

Bacteria were grown in suitable media at different temperatures until advanced stationary phase, then pelleted (7500 rpm for 10 min) and washed once in sterile water. Bacteria were resuspended and transferred to culture flasks at a final 2.10^8^ CFU/ml in 5 ml. Fish larvae were transferred to small Petri dishes to eliminate the chorions and dispatched to the different flasks (15 larvae per flask). After 6 h of incubation with the pathogen at 28°C, individual larvae were distributed into 24-well plates containing 2 ml of water and 50 µL of freshly prepared *T. thermophila* per well to properly monitor their individual fate (1 larva per well). We checked that *E. ictaluri* is not pathogenic to *Tetrahymena*, which are able to feed on *E. ictaluri* bacteria when co-incubated in fish water. Between 6 and 24 larvae were used per condition per experiment. Sterility of control germ-free larvae subjected to mock infection was monitored throughout the experiment by plating and 16S PCR analysis (data not shown). Each experiment was repeated at least 3 times. The larva population was followed on a daily basis and mortality recorded. Dead embryos could be readily identified in microtiter wells as they become opaque. Dubious cases were systematically checked under a stereomicroscope and larvae were considered as dead when complete arrest of all movement, including any heartbeat was observed. Opacification of the larva was always found to follow shortly.

### Probiotic exposure

Probiotic strains were grown for 24 or 48 h in suitable media and temperature. Bacteria were then pelleted and washed once in water. They were diluted at a final concentration of 2.10^7^ CFU/ml. At 4 dpf, just after hatching, zebrafish larvae were put in contact with the probiotic strains by transferring them to probiotic-containing flasks (15 larvae per flask). At 6 dpf, larvae were transferred individually into wells of a 24-well plate containing sterile mineral water inoculated with pathogenic bacteria (2.10^7^ CFU/ml, final concentration). Mortality was followed daily as above. Each experiment was repeated at least 2 times and between 12 and 24 larvae were used per condition per experiment.

### CFU count

Zebrafish were euthanized with tricaine (MS-222) (Sigma-Aldrich #E10521) at 200 mg/ml. Then they were washed in 3 different baths of sterile PBS-0.1% Tween to remove bacteria loosely attached to the skin. Finally, they were transferred to tubes containing calibrated glass beads (acid-washed, 425 µm to 600 µm, SIGMA-ALDRICH #G8772) and 500 µl of autoclaved PBS. They were homogenized using FastPrep Cell Disrupter (BIO101/FP120 QBioGene) for 45 s at maximum speed (6.5 m/s). Finally, serial dilutions of recovered suspension were spotted on plates. CFU were counted after incubation at the appropriate temperature.

### *E. ictaluri* growth and biofilm formation

*Biofilm assay: E. ictaluri* was mixed in a 1∶1 ratio with filtered supernatants of probiotic strains and grown in 96-well microtiter plates at 28°C for 48 h. Microtiter plates were then washed 3 times with water and stained with crystal violet. Biofilm formation was quantified by dissolution of crystal violet and measurement at OD 595 nm. *E. ictaluri growth in presence of probiotic supernants: E. ictaluri* inoculum was mixed in a 1∶1 ratio with filtered supernatant from *E. coli* MG1655, *E. coli* ED1a-sm, and *V. parahaemolyticus* and allowed to grow at 28°. OD 600 nm measurements were taken every 30 minutes. The assay was performed twice in microtiterplates, and 12 different wells were monitored for each condition. *Broth co-cultures of E. ictaluri with the three identified protective strains*. 3 ml of BHI medium was inoculated with *E. ictaluri* alone or with probiotic strain and co-cultures were incubated at 30°C with agitation. Serial dilutions of over-night resulting co-cultures were spotted on BHI+catalase plates in order to obtained isolated colonies (*E. ictaluri* forms patches rather than individualized colonies in absence of catalase). Plates were incubated at 30°C overnight and *E. ictaluri* and *E. coli* MG1655, *E. coli* ED1a-sm, and *V. parahaemolyticus* cfu were counted. *E. ictaluri* was distinguished from co-cultivated bacteria based on its characteristic yellowish colony morphotype. Three co-cultures were tested for each condition.

### Quantitative PCR

Total RNAs from 3–5 pooled zebrafish larvae or a single larva were prepared using Tri-Reagent (Sigma). Oligo(dT_17_)-primed reverse transcriptions were done using M-MLV H- reverse-transcriptase (Promega). Genomic DNA from 3 pooled larvae were prepared using DNeasy blood and tissue kit (Qiagen). Quantitative PCRs were performed using Power SYBR Green PCR Mastermix on an ABI7300 thermocycler (Applied Biosystems). For cDNAs, *Ef1α* was used as a housekeeping gene; for genomic DNA, zebrafish *csf1r* gene was quantified to normalize the amount of DNA. Data were analyzed using the ΔΔCt method as described in [66]. The primers are listed in table S5.

### *In situ* hybridization

WISH was performed using standard zebrafish protocols [65]. To generate the IL-1β antisense probe, IL-1β was amplified by PCR with a T3-modified antisense primer (see Table S5 in text S1). PCR products were purified with Qiaquick PCR purification kit (Qiagen) and the probe was transcribed *in vitro* with T3 polymerase (Promega). Unincorporated nucleotides were removed by purification on NucAway spin columns (Ambion).

### Immunohistochemistry

Whole-mount immunohistochemistry was performed using standard zebrafish protocols [65]. Anesthetized animals were fixed overnight at 4°C in 4% methanol-free formaldehyde (Polysciences, Warrington, PA) in PBS. Permeabilization was performed by a 1 h treatment at room temperature with 1 mg/ml collagenase (Sigma). As primary antibodies, a rabbit polyclonal antiserum that recognizes *E. coli* and a chicken anti-GFP polyclonal were used at a 1∶800 dilution. The secondary antibodies used were Cy3-labeled goat anti-rabbit IgG (Jackson Immunoresearch) diluted 1∶500 and Alexa 488-labeled goat anti-chicken (Invitrogen) diluted 1∶800. The non-specific Cy3 signal observed in the tissues is also observed in the tissues is also observed with other rabbit polyclonal antisera. Nuclei were stained for 30 min at room temperature with Hoeschst 33342 at 2 µg/ml (Invitrogen).

### Imaging

Imaging was performed as described in detail in [67]. Briefly, video-enhanced Nomarski/DIC images of live larvae were taken using a Nikon Eclipse 90i microscope equipped with a Hitachi HV-C20 camera and video captured on miniDV tapes; single frames were later captured using BTVpro software. Images of larvae stained by WISH were taken with a Leica MZ16 stereomicroscope using illumination from above. Images of larvae stained by immunohistochemistry were taken with a fluoIII MZ-16 stereomicroscope (Leica Microsystems, Solms, Germany) equipped with a DS-5Mc camera (Nikon, Tokyo, Japan). Confocal images of live or fixed larvae were taken with a Leica SPE inverted confocal microscope. Confocal images of live or fixed larvae were taken with a Leica SPE inverted confocal microscope equipped with 16× (NA 0,5) and 40× (NA 1,15) oil immersion objectives. Images were processed with the LAS-AF (Leica), ImageJ and Adobe Photoshop softwares.

### Statistical methods

Statistical analyses were performed using one-way analysis of variance (ANOVA) with Bonferroni contrasts unless indicated otherwise. Analyses were performed using Prism v5.0 (GraphPad Software).

### Genes and proteins mentioned in the text

Zebrafish transcripts measured by RT-PCR:

*ef1a* NM_131263

*il1b* BC098597

*tnfa* NM_212859

*il22* NM_001020792

*il10* NM_001020785

Genes targeted for qPCR of genomic DNA:

In zebrafish: *csf1r* gene NM_131672

In *E. ictaluri*: non-coding region next to the *purH* gene – see genomic sequence EU285521

## Supporting Information

Text S1 **This file contains all supporting supplementary materials associated with the presented data.** It includes all supplementary figures (Figures S1, S2, S3, S4, S5, S6) and supplementary tables (Tables S1, S2, S3, S4, S5) along with corresponding legends.
(DOCX)

Video S1 ***Edwardsiella ictaluri*** **colonizes both sides of the lower jaw of zebrafish larvae.** Larva analyzed 3 days post-infection by whole-mount immunofluorescence, using an antibody staining bacteria; fluorescence image (red) superimposed to transmission images (gray). Larva Z-stack taken with a confocal microscope and 40× objective. Ventral view with some lateral tilt, anterior to bottom. The first image of the movies provides a visual help on the top left corner (over the eye) roughly indicating the planes of observation throughout the movie, and a coloured scheme of the cartilages visible in the stack. mc: Meckel's cartilage; pq: palatoquadrate; bh: basihyal; ch: ceratohyal (see Kimmel CB; Miller CT and Moens CB. (2001),Specification and morphogenesis of the zebrafish larval head skeleton. Dev Biol. 15;233(2):239–57.) The yellow line depicts the contour of the fish. Note that figure 3D corresponds to a maximal projection of planes 61 to 75 of the whole stack.
(AVI)
