# Supplementary material for: A New Zebrafish Model of Oro-Intestinal Pathogen Colonization Reveals a Key Role for Adhesion in Protection by Probiotic Bacteria
Source: PLoS Pathog. 2012 Jul 26;8(7):e1002815. doi: 10.1371/journal.ppat.1002815 (PMC3406073; doi:10.1371/journal.ppat.1002815)
Supplement: Text S1 — This file contains all supporting supplementary materials associated with the presented data. It includes all supplementary figures (Figures S1, S2, S3, S4, S5, S6) and supplementary tables (Tables S1, S2, S3, S4, S5) along with corresponding legends. (DOCX) [file ppat.1002815.s001.docx]

SUPPLEMENTARY TABLES

**TABLE S1. Bacterial pathogens used to screen for potential lethal effects in axenic zebrafish larvae.**

| **Name** | **Origin** | **Reference** |
| --- | --- | --- |
| *Aeromonas hydrophila sp. anaerogenes* CIP 76.15 | Used oil-emulsions | CRBIP |
| *Aeromonas hydrophila sp. dhakensis* CIP 107500 | Human feces, Bangladesh | CRBIP |
| *Aeromonas hydrophila sp. hydrophila* CIP 52.94 | Frog | CRBIP |
| *Aeromonas hydrophila sp. hydrophila* CIP 76.14 | Tin of milk with a fishy odor | CRBIP |
| *Aeromonas hydrophila sp. hydrophila* CIP 103561 | Unknown | CRBIP |
| *Aeromonas hydrophila sp. hydrophila* CIP 103697 | Unknown | CRBIP |
| *Aeromonas hydrophila sp. hydrophila* CIP107274 | Human feces | CRBIP |
| *Aeromonas hydrophila sp. hydrophila* CIP 200522 | Fish isolate | CRBIP |
| *Aeromonas hydrophila sp. ranae* CIP 107985 | Frog, Thailand | CRBIP |
| *Aeromonas salmonicida sp. achromogenes* CIP 104001 | Trout, *Salmo trutta*, UK | CRBIP |
| *Aeromonas salmonicida sp. masoucida* CIP 103210 | Fish blood, *Oncorhynchus masou* | CRBIP |
| *Aeromonas salmonicida sp. pectinolytica* CIP 107036 | River water, Argentina | CRBIP |
| *Aeromonas salmonicida sp. salmonicida* CIP 63.4 | Fish, trout | CRBIP |
| *Aeromonas salmonicida sp. salmonicida* CIP 107106 | Diseased fish, Denmark | CRBIP |
| *Aeromonas veronii* CIP 109836 | Fish | CRBIP |
| *Edwardsiella ictaluri* CIP 81.96 | Catfish enteric septicemia, USA | CRBIP |
| *Edwardsiella tarda* CIP 78.61 | Human, feces, USA | CRBIP |
| *Listonella anguillara* CIP 63.36 | Ulcerated cod, *Gadus morhua*, Norway | CRBIP |
| *Listonella anguillara* CIP 64.14 | Ulcerous lesion in plaice, *Pleuronectes platessa*, UK | CRBIP |
| *Listonella anguillara* CIP 73.4 | Diseased rainbow trout | CRBIP |
| *Photobacterium damselae sp. piscicida* CIP 103910 | White perch, USA | CRBIP |
| *Vibrio parahaemolyticus* CIP 71.1 | Sea fish isolate | CRBIP |
| *Vibrio parahaemolyticus* CIP 71.2 | Sea fish isolate | CRBIP |
| *Vibrio ichthyoenteri* CIP 104815 | Japanese flounder fish isolate | CRBIP |
| *Yersinia ruckeri* CIP 82.80 | Rainbow trout, red mouth disease, USA | CRBIP |

**CRBIP:** *Institut Pasteur, Centre de Resources Biologiques de l’Institut Pasteur.*

**TABLE S2. Commensal and probiotic bacteria used to screen for potential protective effect in axenic zebrafish larvae.**

| **Name** | **Origin** | **Reference** |
| --- | --- | --- |
| *Aeromonas veronii* CIP 109836 | Fish | CRBIP |
| *Bacillus cereus* CIPA28 | Lactic ferment | CRBIP |
| *E. coli* BW25113 | Laboratory strain | [[1](#_ENREF_1)] |
| *E. coli* ED1a | Human feces from healthy man (France), Probiotic | [[2](#_ENREF_2)] |
| *E. coli* ED1a-sm | Spontaneous streptomycin-resistant mutant of ED1a | This study |
| *E. coli* ED1a-sm F’*tet* | Laboratory strain | This study |
| *E. coli* K-12 MG1655 | Laboratory strain | [[3](#_ENREF_3)] |
| *E. coli* K-12 MG1655 *attB::gfp-bla* (F’*tet ∆traD::apra ∆tetR::zeo tetA::*Tn*luxCDABE*-Km) | Laboratory strain | This study |
| *E. coli* 10.22 | Commensal strain from hospitalized adult faeces | [[4](#_ENREF_4)] |
| *E. coli* 10.94 | Commensal strain isolated from healthy adult faeces | [[4](#_ENREF_4)] |
| *E. coli* 11.25 | Commensal strain isolated from a healthy adult | [[4](#_ENREF_4)] |
| *E. coli* Nissle 1917 - DSM6601 | Human feces, Commensal strain used as probiotic | [[5](#_ENREF_5)] |
| *E. coli* 083 | Commensal strain used as probiotic | Lab collection |
| *Enterobacter cloacae* 10.91 | Commensal strain isolated from a healthy adult | C. Le Bougennec |
| *Lactobacillus casei* CIP103137 T-ATCC393 | Cheese | CRBIP |
| *Lactobacillus casei sp. rhamnosus* | Human isolate used as probiotic | Lab collection |
| *Lactobacillus delbruecki sp bulgaricus* CIP 101027 | Bulgarian yogurt | CRBIP |
| *Lactobacillus paracasei sp. paracasei* CIP103918 T-ATCC25302 | Unknown | CRBIP |
| *Lactobacillus paracasei* CIP 109805 | Human feces, The Netherlands | CRBIP |
| *Lactobacillus plantarum* WCFS1 | Human saliva, Probiotic | [[6](#_ENREF_6)] |
| *Lactobacillus rhamnosus* A157T-ATCC7449 | Unknown | CRBIP |
| *Lactobacillus rhamnosus* GG – ATCC53103 | Probiotic human isolate | [[7](#_ENREF_7)] |
| *Pediococcus acidilactici* CIP 103408 | Neotype strain | CRBIP |
| *Phaeobacter inhibens* CIP 109852 | Fish, *Scophthalmus maximus*, Spain | CRBIP |
| *Pseudomonas fluorescens* CIP 109851 | Unknown | CRBIP |
| *Vibrio parahaemolyticus* CIP 109835 | Human feces | CRBIP |
| *Escherichia coli* H19 | EHEC UK Human isolate | [[8](#_ENREF_8)] |
| *Escherichia coli* DAEC 7 | Human isolate (Brazil)- Diffusely adhering (DAEC) | [[8](#_ENREF_8)] |
| *Escherichia coli* DAEC18 | Human isolate (Brazil)- Diffusely adhering (DAEC) | [[8](#_ENREF_8)] |
| *Escherichia coli* iai 44 | Human isolate (France)- Urinary infection | [[8](#_ENREF_8)] |
| *Escherichia coli* iai 73 | Human isolate (France)- septicemia | [[8](#_ENREF_8)] |
| *Escherichia coli* G001 | Human isolate (French Guyana) | [[8](#_ENREF_8)] |
| *Escherichia coli* Ec111 | Commensal, Roe-Deer, *Capreolus capreolus,* France | [[8](#_ENREF_8)] |
| *Escherichia coli* Ec029 | Commensal Impala, *Aepyceros melampus* Gabon | [[8](#_ENREF_8)] |
| *Escherichia coli* Ec248 | Commensal, Horse, *Equus caballus*, France | [[8](#_ENREF_8)] |
| *Escherichia coli* Ec300 | Commensal, Dog, *Canis familiaris*, France | [[8](#_ENREF_8)] |
| *Escherichia coli* Ec212 | Commensal, Horse, *Equus caballus*, France | [[8](#_ENREF_8)] |

**CRBIP:** *Institut Pasteur, Centre de Resources Biologiques*

**References**

1. Baba T, Ara T, Hasegawa M, Takai Y, Okumura Y, et al. (2006) Construction of Escherichia coli K-12 in-frame, single-gene knockout mutants: the Keio collection. Mol Syst Biol 2: 2006 0008.

2. Clermont O, Lescat M, O'Brien CL, Gordon DM, Tenaillon O, et al. (2008) Evidence for a human-specific Escherichia coli clone. Environ Microbiol 10: 1000-1006.

3. Guyer MS, Reed RR, Steitz JA, Low KB (1981) Identification of a sex-factor-affinity site in E. coli as gamma delta. Cold Spring Harb Symp Quant Biol 45 Pt 1: 135-140.

4. Hilali F, Ruimy R, Saulnier P, Barnabe C, Lebouguenec C, et al. (2000) Prevalence of virulence genes and clonality in Escherichia coli strains that cause bacteremia in cancer patients. Infection and immunity 68: 3983-3989.

5. Grozdanov L, Raasch C, Schulze J, Sonnenborn U, Gottschalk G, et al. (2004) Analysis of the genome structure of the nonpathogenic probiotic Escherichia coli strain Nissle 1917. Journal of Bacteriology 186: 5432-5441.

6. Kleerebezem M, Boekhorst J, van Kranenburg R, Molenaar D, Kuipers OP, et al. (2003) Complete genome sequence of Lactobacillus plantarum WCFS1. Proc Natl Acad Sci U S A 100: 1990-1995.

7. Kankainen M, Paulin L, Tynkkynen S, von Ossowski I, Reunanen J, et al. (2009) Comparative genomic analysis of Lactobacillus rhamnosus GG reveals pili containing a human- mucus binding protein. Proc Natl Acad Sci USA 106: 17193-17198.

8. Escobar-Páramo P, Le Menac'h A, Le Gall T, Amorin C, Gouriou S, et al. (2006) Identification of forces shaping the commensal Escherichia coli genetic structure by comparing animal and human isolates. Environmental Microbiology 8: 1975-1984.

**TABLE S3. CFU quantification at 9 dpf of germ-free larvae pretreated with selected probiotics at 4 dpf.** Means and standard deviations of the number of CFU recovered from larvae are reported (n=4).

|  | **Cfu/larvae** |
| --- | --- |
| Germ free | 0 ± 0 |
| *E. coli* K-12 MG1655 F’ | 2.2 x10^3^ ± 6.11x10^2^ |
| *E. coli* ED1a | 1.93x10^4^ ± 1.04x10^3^ |
| *V. parahaemolyticus* | 7.15x10^3^ ± 1.08 x10^3^ |
|  |  |

**TABLE S4.  qPCR quantification of colonization by E. ictaluri in germ-free and probiotic-preteated  larvae .** Shown are mean ± SEM from three pools of three larvae each; data have been normalized to one of the samples from high-dose *E. ictaluri*.

|  | ***E. ictaluri gDNA / fish gDNA*** | |
| --- | --- | --- |
| *E. ictaluri* 2.10^6^ 3dpi | | 0.22 ± 0.04 |
| *E. ictaluri* 2.10^7^3dpi | | 0.85 ± 0.31 |
| *E. ictaluri* 2.10^8^ 3dpi | | 1.10 ± 0.19 |
| *E. coli* MG1655 + *E. ictaluri* 2.10^8^ 3dpi | | 1.24 ± 0.49 |
| *E. coli* MG1655F’ + *E. ictaluri* 2.10^8^ 3dpi | | 1.62 ± 0.45 |

**TABLE S5. Primers used in this study***.*

| **Procedure** | **Name** | **Sequence** |
| --- | --- | --- |
| *Sterility-16S* | B27 forward | 5’-AGAGTTTGATCCTGGCTCAG -3’ |
|  | B1492 reverse | 5’-GGTTACCTTGTTACGACTT -3’ |
|  |  |  |
| *IL-1*β *antisense probe* | IL-1β forward | 5’-ATGGCATGCGGGCAATATGA-3’ |
|  | IL-1β reverse | 5’-GAATTCATTAACCCTCACTAAAGGGAGGCCAGGTACAGGTTACTTT-3’ |
|  |  |  |
| *RT-PCR* | TNF-α forward | 5’-CAGAGTTGTATCCACCTGTTA -3’ |
|  | TNF-α reverse | 5’-TTCACGCTCCATAAGACCCA -3’ |
|  |  |  |
|  | IL-1β forward | 5’-GAGACAGACGGTGCTGTTTA -3’ |
|  | IL-1β reverse | 5’-GTAAGACGGCACTGAATCCA -3’ |
|  |  |  |
|  | IL-10 forward | 5’-AGAGCAGGAGAGTCGAATGC -3’ |
|  | IL-10 reverse | 5’-GTACCTCTTGCATTTCACCA -3’ |
|  |  |  |
|  | IL-22 forward | 5’-TTGGAATCAGACGAGCACAC-3’ |
|  | IL-22 reverse | 5’-GGCCAAATCCATAATTGCAC-3’ |
|  |  |  |
| *genomic qPCR* | Eictaluri-forward  forward | 5’-AGCGCCACCTTTGTGGATAA-3’ |
|  | Eictaluri-reverse | 5’-TACGCTTTCCTCAGTGAGTG-3’ |
|  | Csf1r-forward | 5’-TGGACTTCACAGGAACATACAAG-3’ |
|  | Csf1r-reverse | 5’-TCGGAGAAACAAAGAGAACTCG-3’ |

SUPPLEMENTARY FIGURES

***
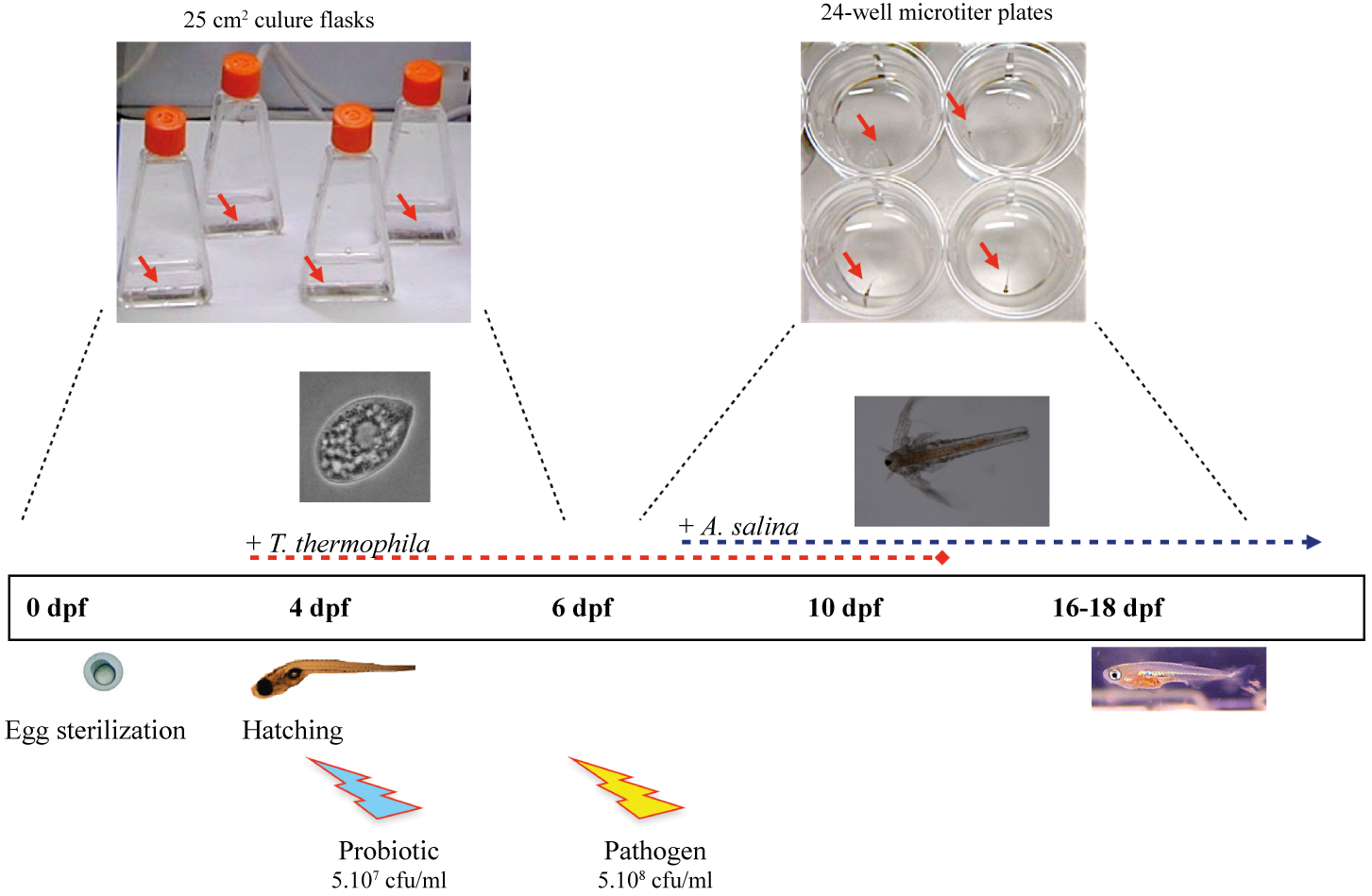
***

**Figure S1,** Rendueles *et al*

**Figure S1. Protocol and timeline of axenic zebrafish infection and co-infection used in this study.** After fertilization, eggs are sterilized and kept in sterile, autoclaved mineral water at 28°C in vented cap cell culture flasks until 6 dpf. Zebrafish larvae are then transferred one-by-one into 24-well microtiter plates containing 2 ml of water per well. Starting at 4 dpf, larvae are fed every 2 days with axenic *T. thermophila* till day 15. For longer experiments, in addition to *Tetrahymena*, larvae were also fed axenic *A. salina* from 10 dpf onwards. Pathogenic bacteria are added to the water at 6 dpf for 6 h and then larvae are transferred to fresh water. To test the protective effect of potentially probiotic strains, larvae were pre-colonized by commensal bacteria diluted in water at 4 dpf, after hatching.


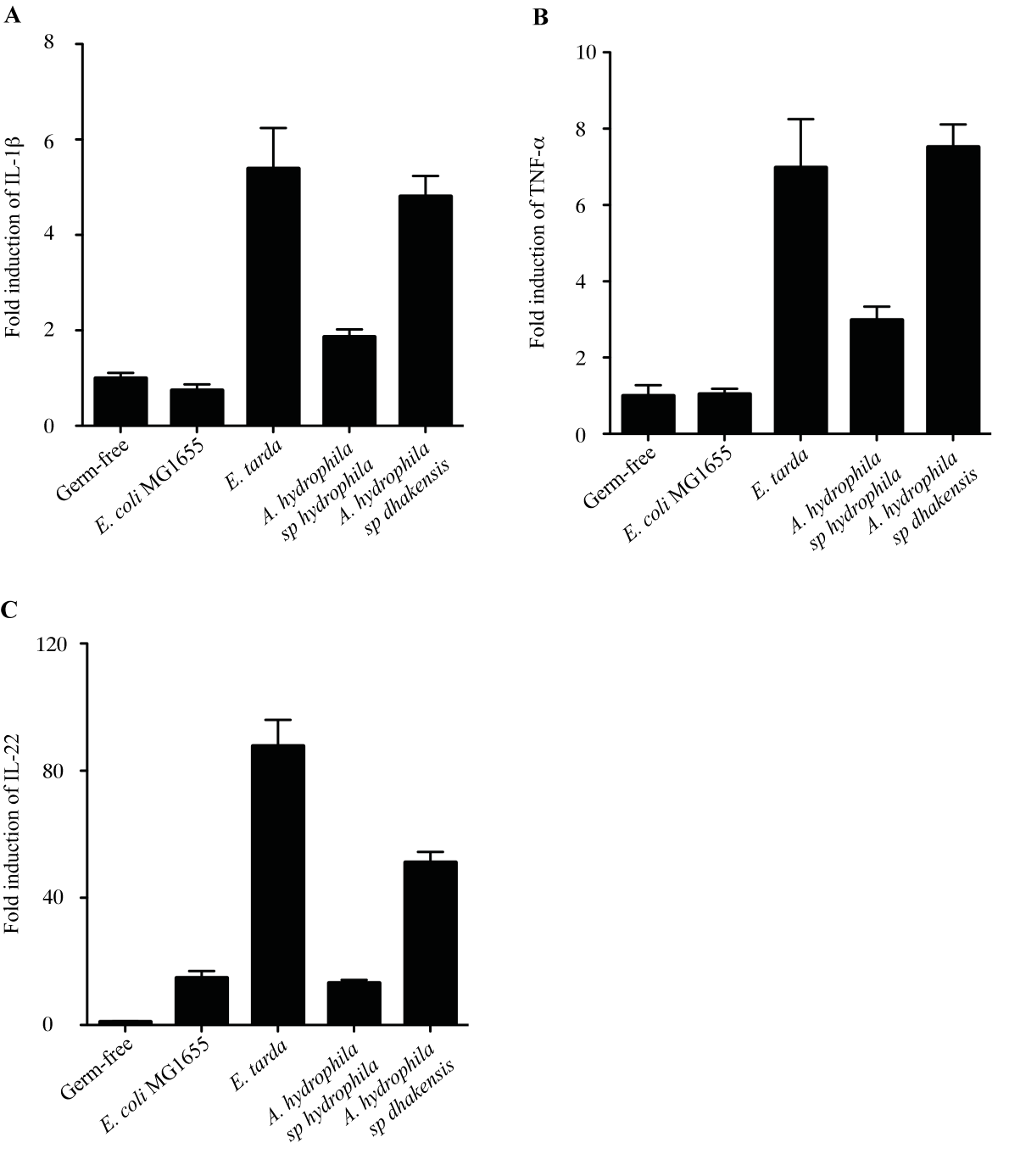


**Figure S2,** Rendueles *et al*

**Figure S2. Inflammation marker expression of gnotobiotic zebrafish larvae upon infection by mild pathogens.** qRT-PCR was performed using primers specific to *il1b* (**A**), *tnfa* (**B**), and *il22* (**C**) (inflammation markers) on RNA extracted from pools of 5 larvae at 3 dpi from germ-free larvae or larvae exposed to *E. coli* MG1655 (control), *E. tarda, A. hydrophila* sp. hydrophila or *A hydrophila* sp. *dhakensis* at 4 dpf. Levels are expressed relative to the germ-free larvae. Error bars represent 95% confidence intervals from three technical replicates; one representative experiment out of two.


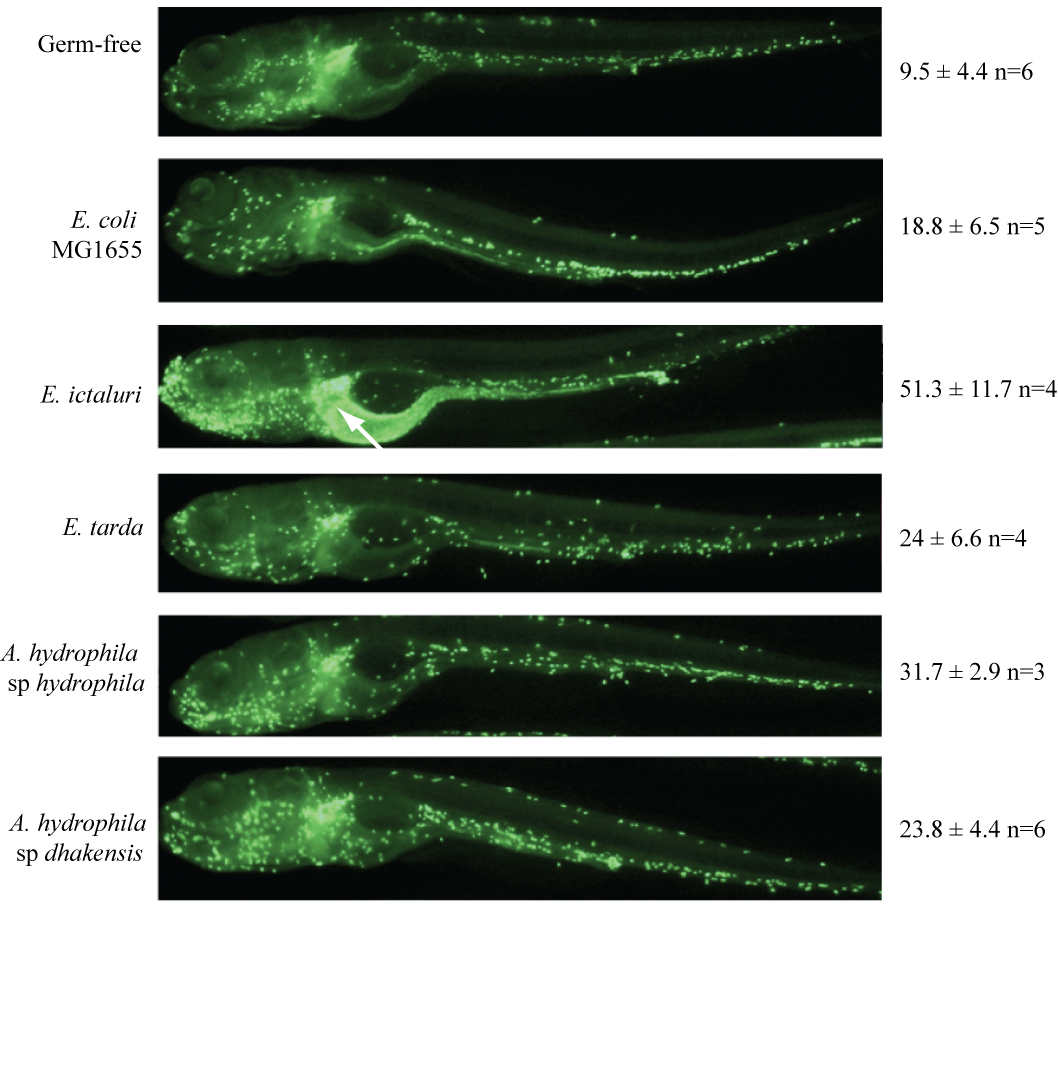


**Figure S3,** Rendueles *et al*

**Figure S3. Neutrophil localization upon pathogen infection**. Neutrophil infection in germ-free *mpx::gfp* larvae or *mpx::gfp* larvae infected with *E. coli* MG1655 (control) or different pathogens. At 4 days post-infection, larvae were fixed and analyzed by whole-mount immunofluorescence. Neutrophils were detected as GFP-expressing leukocytes (green). A quantification of gut-associated neutrophils in infected larvae is indicated on the right for each group of larvae. Note also GFP-expressing enterocytes as indicated by white arrow.


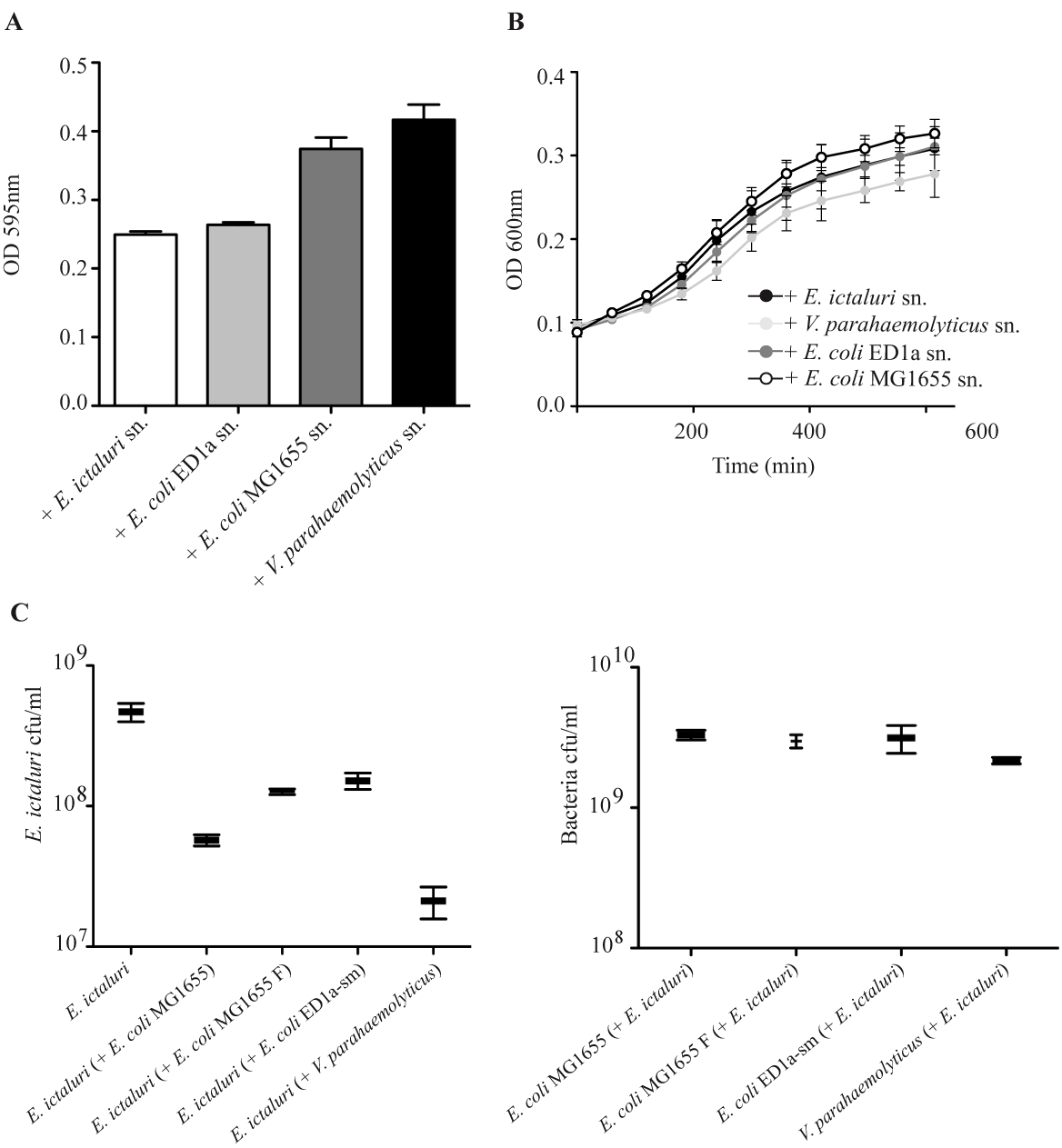


**Figure S4,** Rendueles *et al*

**Figure S4.** **Impact of identified protective strains on *E. ictaluri* growth and biofilm formation**  (**A**) Biofilm assay: *E. ictaluri* was mixed in a 1:1 ratio with filtered supernatants of probiotic strains and grown in 96-well microtiter plates at 28°C for 48 h. Microtiter plates were then washed 3 times with water and stained with crystal violet. Biofilm formation was quantified by dissolution of crystal violet and measurement at OD 595 nm. Addition of *E. ictaluri*’s own supernatant was included as a control. (**B**) *E. ictaluri* growth in presence of probiotic supernants: *E. ictaluri* inoculum was mixed in a 1:1 ratio with filtered supernatant (sn.) from *E. coli* MG1655*,E. coli* ED1a-sm, and *V. parahaemolyticus* and allowed to grow at 28°. OD 600 nm measurements were taken every 30 minutes. Growth of *E. ictaluri* with its own supernatant was included as a control. The assay was performed twice in microtiterplates, and 12 different wells were monitored for each condition. (**C**) Broth co-cultures of *E. ictaluri* with the three identified protective strains. 3ml of BHI medium was inoculated with *E. ictaluri* alone or with probiotic strain and co-cultures were incubated at 30°C with agitation. Serial dilutions of over-night resulting co-cultures were spotted on BHI+catalase plates in order to obtained isolated colonies (*E. ictaluri* forms patches rather than individualized colonies in absence of catalase). Plates were incubated at 30°C overnight and *E. ictaluri* and *E. coli* MG1655*,E. coli* ED1a-sm, and *V. parahaemolyticus* cfu were counted. *E. ictaluri* was distinguished from co-cultivated bacteria based on its characteristic yellowish colony morphotype. **Left panel:** *E. ictaluri*  cfu in corresponding co-cultures. **Right panel:** Corresponding protective bacteria cfu in corresponding co-cultures with *E. ictaluri*. Results are expressed as mean±SD of three co-cultures for each condition.

**
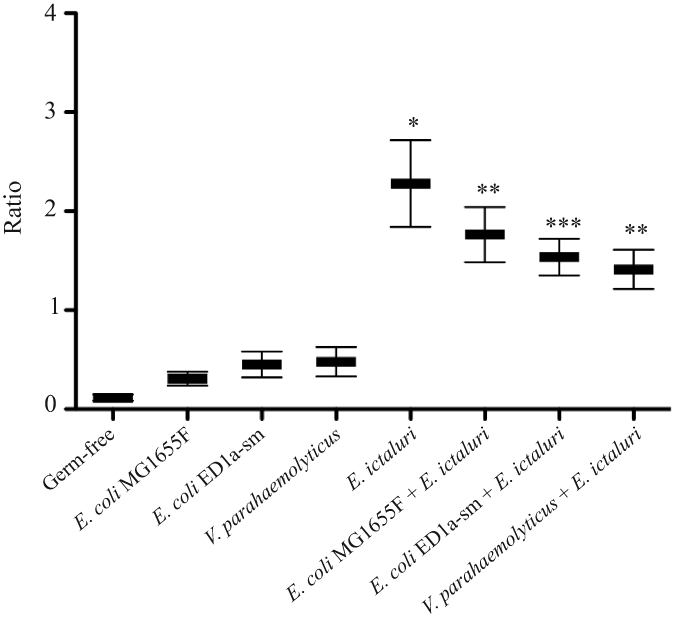
**

**Figure S5,** Rendueles *et al*

**Figure S5*.*Neutrophils redistribution in head and gut.** Neutrophils redistribution was quantified by calculating the ratio (neutrophils counts in hematopoietic sites/neutrophils counts in the head and gut) for each larvae. Results are presented as mean+SEM. Statistical significance was calculated between the corresponding pretreated larvae infected and non-infected by *E. ictaluri* using unpaired two-tailed t-test with Welch's correction (*p<0.05, **p< 0.01, ***p<0.001). One larvae infected by *E. ictaluri* with a high ratio value was considered as an outlier and excluded from this analysis.
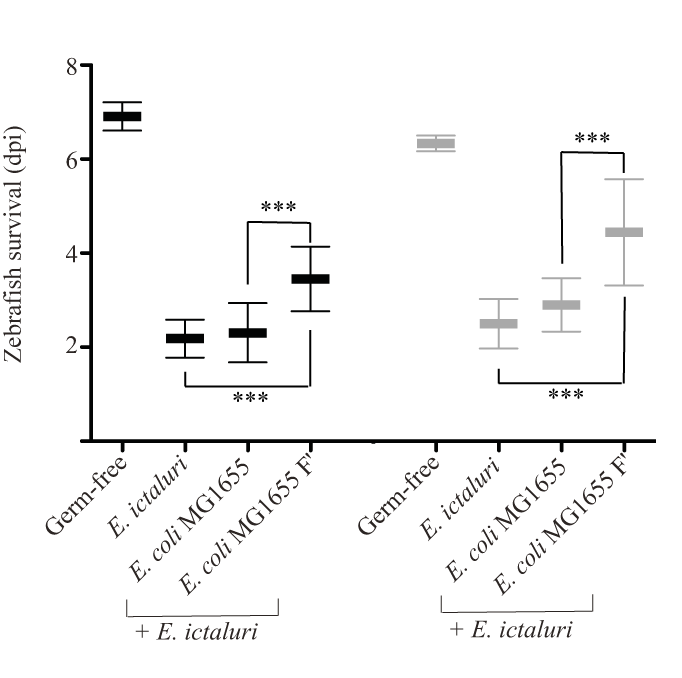


**Figure S6,** Rendueles *et al*

**Figure S6*.*  Compared life expectancy upon *E. ictaluri* infection in germ-free and conventional larvae pre-colonized with various *E. coli*.** Mortality of germ-free (black) and conventional (grey) zebrafish larvae pre-colonized at 4 dpf with *E. coli* MG1655 and *E. coli* MG1655 F’, and infected at 6 dpf with *E. ictaluri*. Mean survival is represented by a large hyphen. Standard deviations are also indicated ( ***p<0.001).

**
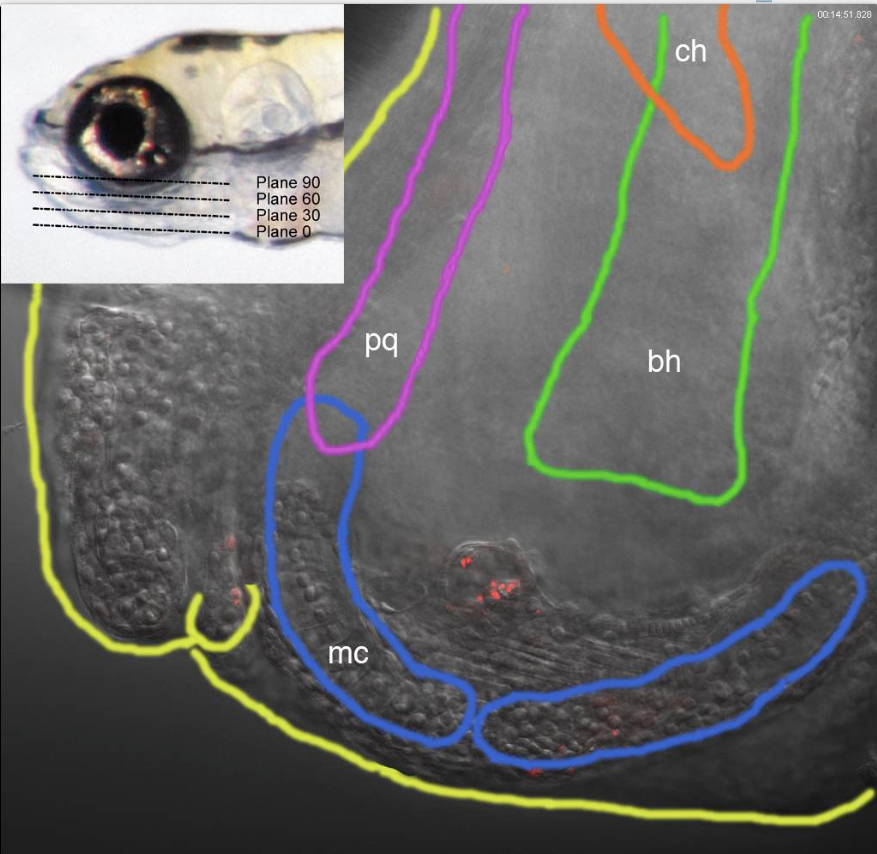
**

**Video S1,** Rendueles *et al*

**Video S1.** ***Edwardsiella ictaluri* colonizes both sides of the lower jaw of zebrafish larvae.** Larva analyzed 3 days post-infection by whole-mount immunofluorescence, using an antibody staining bacteria; fluorescence image (red) superimposed to transmission images (gray). Larva Z-stack taken with a confocal microscope and 40x objective. Ventral view with some lateral tilt, anterior to bottom. The first image of the movies provides  a visual help on the top left corner (over the eye) roughly indicating the planes of observation throughout the movie, and a coloured scheme of the cartilages visible in the stack. mc: Meckel's cartilage; pq: palatoquadrate; bh: basihyal; ch: ceratohyal (see [Kimmel CB](http://www.ncbi.nlm.nih.gov/pubmed?term=%22Kimmel%20CB%22%5BAuthor%5D); [Miller CT](http://www.ncbi.nlm.nih.gov/pubmed?term=%22Miller%20CT%22%5BAuthor%5D) and [Moens CB](http://www.ncbi.nlm.nih.gov/pubmed?term=%22Moens%20CB%22%5BAuthor%5D). (2001),Specification and morphogenesis of the zebrafish larval head skeleton. Dev Biol. 15;233(2):239-57.) The yellow line depicts the contour of the fish. Note that figure 3D corresponds to a maximal projection of planes 61 to 75 of the whole stack.
